# Transcriptome Analysis Reveals Putative Genes Involved in Iridoid Biosynthesis in *Rehmannia glutinosa*

**DOI:** 10.3390/ijms131013748

**Published:** 2012-10-23

**Authors:** Peng Sun, Shuhui Song, Lili Zhou, Bing Zhang, Jianjun Qi, Xianen Li

**Affiliations:** 1 Institute of Medicinal Plant Development, Peking Union Medical College, Chinese Academy of Medical Sciences, Beijing 100193, China; E-Mails: pengsun@126.com (P.S.); llzhou@implad.ac.cn (L.Z.); jjqi@implad.ac.cn (J.Q.); 2 CAS Key Laboratory of Genome Sciences & Information, Beijing Institute of Genomics, Chinese Academy of Sciences, Beijing 100029, China; E-Mails: songshh@big.ac.cn (S.S.); zhangbing@big.ac.cn (B.Z.)

**Keywords:** *Rehmannia glutinosa*, iridoid biosynthesis, transcriptome

## Abstract

*Rehmannia glutinosa*, one of the most widely used herbal medicines in the Orient, is rich in biologically active iridoids. Despite their medicinal importance, no molecular information about the iridoid biosynthesis in this plant is presently available. To explore the transcriptome of *R. glutinosa* and investigate genes involved in iridoid biosynthesis, we used massively parallel pyrosequencing on the 454 GS FLX Titanium platform to generate a substantial EST dataset. Based on sequence similarity searches against the public sequence databases, the sequences were first annotated and then subjected to Gene Ontology (GO) and Kyoto Encyclopedia of Genes and Genomes (KEGG) based analysis. Bioinformatic analysis indicated that the 454 assembly contained a set of genes putatively involved in iridoid biosynthesis. Significantly, homologues of the secoiridoid pathway genes that were only identified in terpenoid indole alkaloid producing plants were also identified, whose presence implied that route II iridoids and route I iridoids share common enzyme steps in the early stage of biosynthesis. The gene expression patterns of four prenyltransferase transcripts were analyzed using qRT-PCR, which shed light on their putative functions in tissues of *R. glutinosa*. The data explored in this study will provide valuable information for further studies concerning iridoid biosynthesis.

## 1. Introduction

*Rehmannia glutinosa*, a well-known medicinal plant from the *Scrophulariaceae* family, has been extensively used in clinics in the Orient to replenish vitality, to treat a variety of ailments such as diabetes, constipation, urinary tract problems, anemia, dizziness, and for regulating menstrual flow [1]. *R. glutinosa* is rich in iridoids. Previous phytochemical investigations on *R. glutinosa* have led to the isolation of diverse compounds, the majority of which are iridoids such as catalpol, aucubin, rehmannioside A, B, C and D; more than 30 in total [2]. The main active principal of *R. glutinosa*, catalpol, was reported to make up 2.7%–10.6%, with an average of 6.3% of the fresh root content of *R. glutinosa* [3]. Intensive studies revealed that iridoids exhibit a wide range of bioactivity such as neuroprotective, antitumor, antinflammatory and antioxidant effects, *etc*. [4]. Catalpol has hypoglycemic, diuretic, laxative effects [2], and recently also demonstrated neuroprotective effects [5–7]. Other iridoids such as aucubin and geniposide in *R. glutinosa* have also been shown to possess biological activities [4].

**Figure 1:**
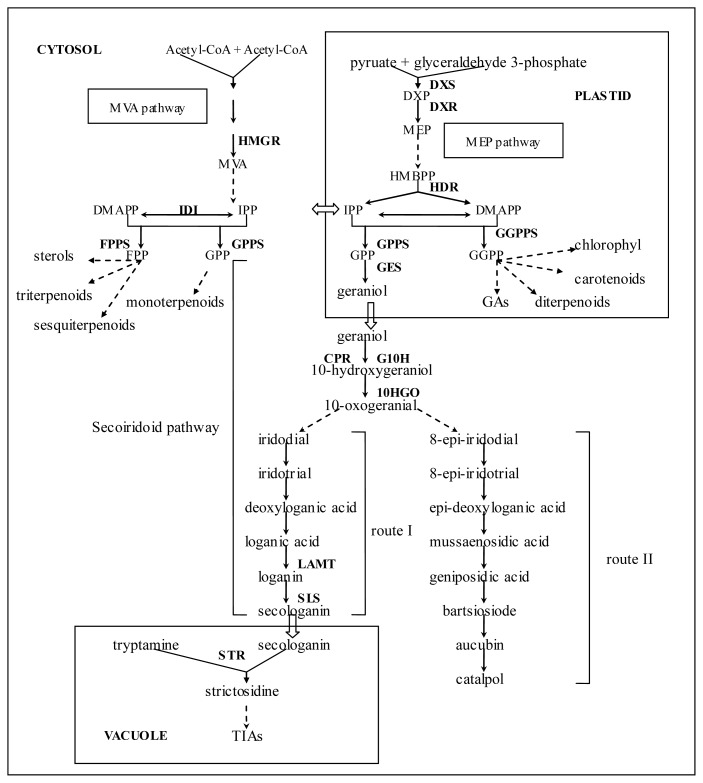
Overview of iridoid biosynthesis in plants. CPR: cytochrome P450 reductase; dmapp: dimethylally diphosphate; DXP: 1-deoxy-d-xylulose 5-phosphate; DXS: 1-deoxy-d-xylulose-5-phosphate synthase; DXR: 1-deoxy-d-xylulose-5-phosphate reductoisomerase; FPP-farnesyl diphosphate; FPPS-farnesyl diphosphate synthase; GPP: geranyl diphosphate; GPPS: geranyl diphosphate synthase; GES: geraniol synthase; GGPP: geranylgeranyl diphosphate; GGPPS: geranylgeranyl diphosphate synthase; G10H: geraniol 10-hydroxylase; 10HGO: 10-hydroxygeraniol oxidoreductase; HMBPP: 1-Hydroxy-2-methyl-2-butenyl-4 diphosphate; HMGR: 3-hydroxy-3-methylglutaryl-CoA reductase; IPP: ispentenyl diphosphate; IDI: IPP isomerase; LAMT: sadenosyl-l-methionine: loganic acid methyl transferase; MEP: 2-*C*-methyl-d-erythritol 4-phosphate; MVA: mevalonic acid; SLS: secologanin synthase; STR: strictosidine synthase; Route I: the route leading to the biosynthesis of secologanin, Route II: the route leading to the biosynthesis of catalpol.

Iridoids represent a large group of monoterpenoids characterized by a functionalized cis-fused cyclopentan-[c]-pyran skeleton. Oxidative cleavage of the cyclopentane ring gives rise to a subclass known as secoiridoids, which are often intermediates in the biosynthesis of terpenoid indole alkaloids (TIA). The biosynthesis of iridoids has been fairly well studied through precursor feeding experiments [8–11]. It is known that two main routes exist for iridoid biosynthesis: route I is from iridodial via deoxyloganic acid and loganin to secologanin, which is the precursor of the derived secoiridoids and complex TIAs; route II involves 8-epi-iridodial, 8-epi-iridotrial and 8-epi-deoxyloganic acid, which are precursors of the decarboxylated carbocyclic iridoids, such as aucubin and catalpol (Figure 1) [8,12]. Over the past two decades, *Catharanthus roseus* has been the best investigated species in the field of iridoid biosynthesis providing more than one hundred medicinally important TIAs, such as the anti-cancer compounds vinblastine and vincristine. The iridoid moiety of TIA belongs to the secoiridoid subclass that is derived from iridoid biosynthetic route I, which is regarded as rate limiting for TIA production [13–15]. The secoiridoid pathway (sometimes also called “iridoid pathway”) encompasses the biosynthetic route I and several upper steps that involve GPP, geraniol, 10-hydroxygeraniol and 10-oxogeranial [14,15]. Intensively biochemical and genetic studies around the *C. roseus* TIA biosynthesis have established the molecular basis of the secoiridoid pathway, and a set of genes involved in this pathway have been cloned and characterized [16–24]. The secoiridoid pathway in *C. roseus* has been well reviewed [14,15]. However, no genetic or molecular study on iridoid biosynthesis has been reported in non-TIA producing plants, and no molecular information about the biosynthesis of route II iridoids is presently available.

*R. glutinosa* is characterized by the presence of catalpol, the most extensively investigated active ingredient in this species that has been shown to possess important pharmaceutical activities [2–6]. The catalpol biosynthetic route was established by Damtoft in 1994 through feeding experiments, which starts from 8-epi-iridodial, via 8-epi-deoxyloganic acid, bartsioside, and aucubin to catalpol (Figure 1) [11], namely, the iridoid biosynthetic route II termed by Jensen [12]. The upper steps of catalpol biosynthetic pathway have not been established experimentally, but it is suggested that catalpol and other iridoids generated through route II are probably derived from geraniol [8]. In this study, in order to identify genes involved in iridoid biosynthesis, a cDNA library generated from the tuberous root of *R. glutinosa*, which accumulates a high amount of catalpol, was sequenced using the 454 GS FLX Titanium platform and 58,822 unique assembled sequences were obtained. Bioinformatic analysis indicated that all candidate genes involved in terpenoid backbone biosynthesis were within the 454 assembly. Furthermore, we also discovered several genes that encode putative enzymes catalyzing the early enzyme steps of the secoiridoid pathway, including geraniol synthase (GES), geraniol 10-hydroxylase (G10H), cytochrome P450 reductase (CPR) and 10-hydroxygeraniol oxidoreductase (10HGO), which are also very likely to be involved in the early steps leading to route II iridoids. The genes described in this study constitute an important resource for future iridoid biosynthesis studies and increase the practical potential of molecular engineering of the iridoid pathway in this plant. In this study, the “iridoid pathway” is referred to as the catalpol biosynthetic pathway.

## 2. Results and Discussion

### 2.1. Sequence Generation and Function Analysis

A half plate 454 run yielded 88.4 MB bases from 374,444 reads with an average 236 bp. The sequences were deposited at NCBI under the accession number SRX128593. After trimming the adapter sequences and removing low quality sequences, a total of 341,170 clean reads were assembled. The assembly was carried out using CAP3; assembly of the trimmed, size-selected sequences generated a total of 58,822 unique sequences including 21,504 contigs and 37,318 singletons. The average contig length was 397 bases with an average of 14.1 reads assembled per contig. To date, only a small number of rehmannia ESTs have been deposited in GenBank, alignment analysis showed that the majority of ESTs can be found in our 454 sequence collection.

The annotation was based on sequence similarity searches against the known databases, *i.e.*, the NCBI non-redundant protein (Nr) database, the Universal Protein Resource (UniProt) database, the Kyoto Encyclopedia of Genes and Genomes (KEGG) database, the Clusters of Orthologous Groups of proteins (COG) database and the NCBI non-redundant nucleotide (Nt) database, respectively, with an e-value cutoff at e-5. Due to lack of genome and EST information, only 49.0% of the unique sequences were annotated. The annotation summary is shown in Table 1. In order to distinguish redundant sequences from homologous sequences, “unigene” was used in this study to minimize redundancy, each unique sequence was assigned a unigene ID according to the accession number of the best-hit homologue in the Nr database; finally 23,953 unigenes were obtained.

**Figure 2:**
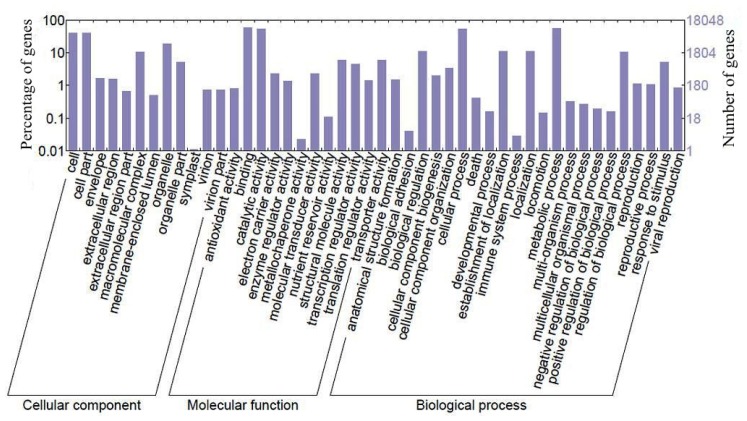
Gene Ontology analysis of genes in the 454 sequence collection of *R. glutinosa*.

**Figure 3:**
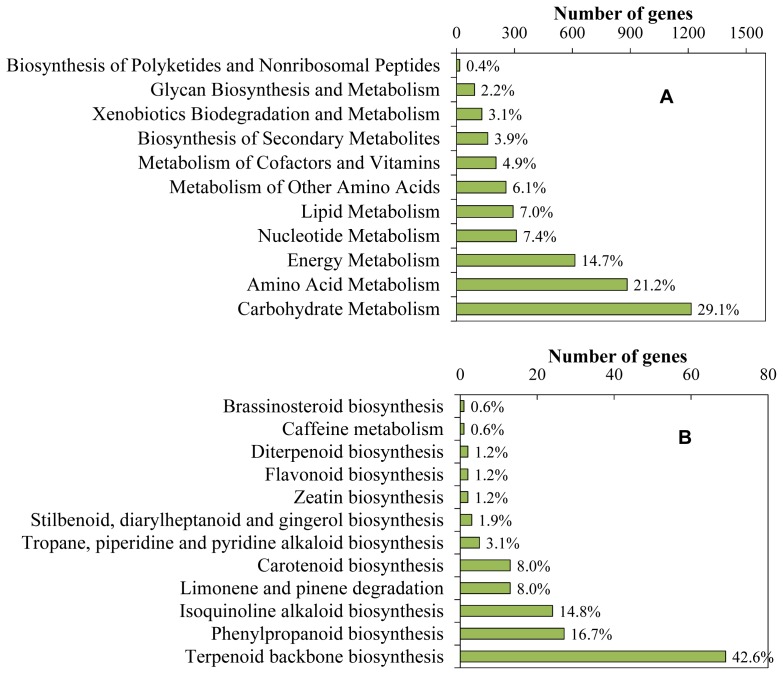
Pathway assignment based on Kyoto Encyclopedia of Genes and Genomes (KEGG). (**A**) Classification based on metabolism categories; (**B**) Classification based on secondary metabolism categories.

Based on Gene Ontology (GO) classifications, 25,468 sequences represented by 46 functional groups were classified into three main GO categories (Figure 2). Among cellular components, cell (40.6%) and organelle (18.7%) categories represented the most dominant groups. Within the molecular function category, binding represented the most abundant category (59.6%) followed by catalytic activity (39.8%) and transport activity (5.9%). A total of 794 unique sequences (4.4%) were designated as transcriptional regulators, with the majority (480) being subcategorized as “transcription factor activity”. Transcription factors play a significant role in the regulation of secondary metabolite biosynthesis by controlling gene expression [25]. In *C. roseus*, overexpression of the jasmonate-responsive transcription factor ORCA3 can enhance TIA production and activate the expression of iridoid biosynthesis related genes [26]. Interestingly, an ORCA3 homologue (contig1996) was also found in the sequence collection. For biological processes, the majority of sequences were grouped into metabolic (56.9%) and cellular process (52.0%). Compared with the sweet potato tuberous root, in which only 30.46% genes were classified into metabolic process [27], the tuberous root of *R. glutinosa* contains a higher percentage of metabolic genes, indicating that more metabolic activities occur in *R. glutinosa*. KEGG analysis provides an alternative functional annotation of genes. Of the unique sequences, 25,258 (42.9%) sequences have sequence similarities to KEGG database. Among them, 4,178 unique sequences having enzyme commission (EC) were assigned to metabolic pathways. As shown in Figure 3A, the metabolic pathways were well represented by carbohydrate metabolism, amino acid metabolism, energy metabolism, and nucleotide metabolism, showing that primary metabolism is vital to the tuberous root growth and development. Within the secondary metabolism category, terpenoid backbone biosynthesis, phenylpropanoid biosynthesis and isoquinoline alkaloid biosynthesis were prominently represented; the genes involved in terpenoid backbone biosynthesis constitute the largest group, accounting for 42.6% of the total genes assigned to this category, showing that the terpenoid biosynthesis occupies a distinguished position among the secondary metabolic activities in *R. glutinosa* (Figure 3B). In this study, by searching the annotation information against the Nr, KEGG, UniProt, COG, GO and Nt database, we totally identified 154 unique sequences involved in primary and central terpenoid biosynthesis, which are members of 33 gene families (Supplementary Files 1 and 2). Among them, the annotations of the putative iridoid pathway genes and short-chain prenyltransferase genes were further verified manually through searching against the protein sequences of reference species deposited in the Nr database and the annotation and alignment information was shown in Supplementary File 3.

### 2.2. Identification of Putative Genes Related to Iridoid Biosynthesis

Iridoids are derived from ispentenyl diphosphate (IPP) and its allylic isomer dimethylally diphosphate (DMAPP) through multiple steps. In higher plants, IPP itself can be formed through either the plastidial 2-*C*-methyl-d-erythritol-4-phosphate (MEP) pathway or the cytosolic mevalonic acid (MVA) pathway. Although experiments indicated that there exists a crosstalk between the MVA and MEP pathways in the terpenoid biosynthetic network in some species, it is well established that MEP pathway mainly leads to monoterpenoids, diterpenoids, the prenyl side chains of chlorophyls and carotenoids, as well as to the phytohormones abscisic acid, cytokinin and gibberellins, and the MVA pathway predominantly gives rise to sterols, sesquiterpenoids and ubiquinones [28]. In TIA producing plants, through precursor feeding experiments, it had been confirmed that the secoiridoids are derived directly from the MEP pathway rather than MVA pathway [29–31]. The crucial role of the MEP pathway in TIA biosynthesis have also been demonstrated at molecular level, the expressions of the three MEP pathway genes, *1-Deoxy-d-xylulose-5-phosphate synthase* (*DXS*), *1-Deoxy-d-xylulose-5-phosphate reductoisomerase* (*DXR*) and *2-*C*-methyl-d-erythritol 2,4-cyclodiphosphate synthase* (*MECS*) in *C. roseus* are consistent with the TIA production [17,18,23,24]. Based on the gene annotation information, we identified all MEP pathway genes. Among them, the transcripts of DXS and DXR were represented by many 454 reads, indicating that they are highly expressed in the tuberous roots of *R. glutinosa*. Interestingly, genes encoding all enzymes in MVA pathway were also found in the 454 dataset, especially the transcripts of 3-hydroxy-3-methylglutaryl-CoA reductase (HMGR) were represented by more than one hundred 454 reads, which seemed to show that the MVA pathway plays important roles in tuberous roots (Table 2). Present data cannot exclude the participation of the MVA pathway to the biosynthesis of route II iridoids, as evidences from *C. roseus* showed that the MVA pathway may be implicated in the regulation of MEP and secoiridoid pathway genes through protein prenylation [32]. Interestingly, a set of genes encoding putative farnesyltransferases were found in our sequence dataset (data not shown). However, the MEP pathway will be the major source of precursors (IPP and DMAPP) for the iridoid biosynthesis as it is in other monoterpenoid producing plants. The presence of high amount of MVA pathway gene transcripts is most likely to show that other terpenoids exist, derived from this pathway, for example, the biosynthesis of sterols and other triterpenoids that are generally derived from the MVA pathway. A relatively large amount of genes involved in the biosynthesis of other terpenoids, including steroid biosynthetic genes, had also been identified in the datasets (Supplementary File 1).

Most interestingly, putative genes encoding enzymes of the secoiridoid pathway including GES, G10H, CPR and 10HGO were found in the 454 dataset. Among these enzymes, G10H and 10HGO have only been identified in TIA producing plants. The alignment results showed that these genes share significant sequence similarities with the secoiridoid pathway genes (Supplementary File 3). The predicted amino acid sequences of the putative *R. glutinosa G10H* gene (contig14037) are 80% identical to those of CrG10H (Supplementary File 4), which implied that it may catalyze the hydroxylation of geraniol as CrG10H does in *C. roseus*. It is known that the early steps of secoiridoid pathway involve GPP, geraniol, 10-hydroxygeraniol and 10-oxygerania, enzymes catalyzing above steps have been characterized (Figure 1). The first enzymatic step in the secoiridoid pathway is catalyzed by GES, which converts GPP into geraniol. The enzyme and its encoding gene were first characterized from the peltate glands of sweet basil [33]. In the next step, G10H hydroxylates geraniol to form 10-hydroxygeraniol. G10H is a cytochrome P450 monooxygenase belonging to the CYP76B superfamily, which controls the first committed step in the biosynthesis of TIAs in *C. roseus* [20,21]. CPR which is essential for the G10H catalyzed reaction presumably acts as the electron donor for G10H [18]. The gene expression profiles of the CPR and G10H of *C. roseus* are similar and they respond to jasmonic acid induction with similar kinetics [20]. The conversion of 10-hydroxygeraniol to 10-oxogeranial is carried out by 10HGO, a NADP+ oxidoreductase. This enzyme was first purified and characterized in *Rauwolfia serpentina* [34]. So far, the early steps of the iridoid pathway have not been detailed, though they were suggested to be similar to those of the secoiridoid pathway [8]. As we know, no secoiridoid compound has been identified in *R. glutinosa*, oppositely, more than 30 iridoids have been isolated from this species. The presence of the high homologues of secoiridoid pathway genes in non-TIA producing plant *R. glutinosa* implied that they are most likely to be implicated in the biosynthesis of route II iridoids and built the molecular base in support of the previous suggestion. So it would be reasonable to conclude that the biosynthesis of iridoids and secoiridoids shares the common enzyme steps before the formation of iridane skeleton (Figure 1).

A comparison of our data with that reported by Sun *et al.* dealing with the pyrosequencing of the transcriptome of *Camptotheca acuminate* [35], an important plant producing TIAs, indicated that our 454 dataset contains more genes involved in iridoid biosynthesis, including the genes *4-diphosphocytidyl-2-C-methyl-d-erythritol synthase* (*CMS*), *geranyl diphosphate synthase* (*GPPS*) and *geraniol synthase* (*GES*) that had not been discovered in Sun *et al.*’s research. In this study, a total of 550 EST sequences representing 21 gene families putatively involved in iridoid biosynthesis were discovered (Table 2). These results demonstrated that our 454 data pool is a good resource for studying the iridoid biosynthesis. The above genes identified in this study constitute the putative candidate iridoid biosynthetic genes and are worthy of in-depth study in future. Most significantly, this 454 dataset is very likely to contain the remaining uncharacterized biosynthetic and regulatory genes of the iridoid pathway. The sequence dataset can be used to identify, and functionally characterize genes involved in the subsequent iridoid skeleton modification, such as cyclization, glycosylation, decarboxylation, hydroxylation, epoxidization, and so on.

### 2.3. Identification of Genes Involved in Biosynthesis of Other Terpenoids

Besides genes related to iridoid biosynthesis, the 454 sequence assembly also contains a number of genes encoding enzymes involved in the formation of other terpenoids, among them, the transcripts involved in biosynthesis of diterpenoids, triterpenoids and their derivatives, such as farnesyl diphosphate synthase (FPPS) and geranylgeranyl diphosphate synthase (GGPPS), are well represented (Supplementary File 1). In plants, diterpenoids form the basis for biologically important compounds such as retinal, phytol, carotenoids and gibberellins, which display important functional roles; triterpenoids are precursors to saponins and steroids, the latter are components of membranes in most organisms. Among these genes, the genes that encode enzymes phytoene synthase (PSY), geranylgeranyl reductase (GGR) and squalene synthase (SQS) are located at the major branch points of the central terpenoid pathway and occupy a particular position in control of product distributions. PSY is a key enzyme of the carotenoid biosynthetic pathway that has also been shown to be involved in ABA formation under abiotic stresses [36]. GGR reduces free geranylgeranyl diphosphate (GGPP) to phytil diphosphate, which provides the side chain to chlorophyls, tocopherols, and plastoquinones [37,38]. SQS, which catalyzes a reductive dimerization of two farnesyl diphosphate (FPP) molecules into squalene, is a key enzyme capable of diverting carbon flow specifically to the biosynthesis of sterols [39]. Four unigenes assembled from 162 reads were annotated as SQS, indicating that they are highly expressed in the tuberous root (Supplementary File 1). Apart from SQS, a relatively large amount of enzyme sequences representing five enzyme families were mapped to the steroid biosynthetic pathway according to the standard KEGG pathway, which implied that a large portion of triterpenoids are channeled towards the steroid pathway. The sequences and annotation information explored in this study will also be a good resource for understanding the biosynthesis of other terpenoids in *R. glutinosa*.

### 2.4. Expression Patterns of Four *Short-Chain Prenyltransferase* Genes

In nature, terpenoid biosynthesis is regulated at multiple metabolic branch points to create large structurally and functionally diverse compounds [40,41]. The so called “short-chain prenyltransferases”, *i.e.*, the enzymes geranyl diphosphate synthase (GPPS), FPPS and GGPPS that catalyze the condensations of IPP and DMAPP to geranyl diphosphate (GPP), FPP and GGPP respectively, are situated at the primary branch point of the central terpenoid pathway, directing carbon flux into different classes of terpenoids and so in control of the product distribution [41]. GPP is the entry point leading to the biosynthesis of all monoterpenoid products; FPP is a key precursor in the formation of sesquiterpenoids and triterpenoids and in the biosynthesis of sterols, brassinosteroids and ubiquinones; while GGPP is a central precursor for a diverse group of primary and specialized terpenoid compounds such as carotenoids, chlorophylls, ABA, gibberellins and diterpenoids [42].

**Figure 4:**
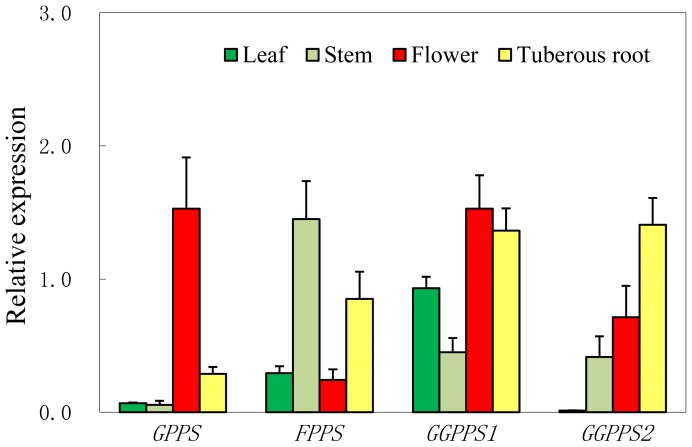
The expression pattern of four selected short-chain prenyltransferase genes in tissues of *R. glutinosa*. GPP: geranyl diphosphate synthase; FPPS: farnesyl diphosphate synthase; GGPPS: geranylgeranyl diphosphate synthase; Means ± SE; Three replicates of each qRT-PCR were performed.

As described above, GPPS is the key branchpoint enzyme leading to monoterpene biosynthesis, so it is valuable to discover *R. glutinosa* GPPS genes and gain an insight into their molecular characters. Among the terpenoid biosynthetic genes identified in this study, we found two sequences whose encoding proteins have high similarities to the *Antirrhinum majus* GPPS large subunit (GPP.LSU), sharing 95% (63/66, ratio of identical amino acids) and 92% (35/38) sequence identities, respectively, which are very likely to belong to the same gene, because they correspond to one unigene. Apart from these, no other putative gene encoding GPPS was discovered. In comparison, the transcripts that were predicated to be *FPPSs* and *GGPPS*s were much more abundant. GPPSs can exist as heterodimeric or homodimeric proteins. In the heterodimeric enzymes, the catalytic GPP.LSU had high amino acid sequence identity to plant GGPPSs, whereas the sequence of the noncatalytic smaller subunit (GPPS.SSU) is related to, but much more divergent from, the sequences of GGPPSs; GPP.LSU can be either an active GGPPS or an inactive GGPPS-like protein, it is the GPP.SSU that determines the product specificity of the catalytic large subunit [43–46]. It had been further shown that GPP.SSU can bind to a variety of bona fide GGPPS enzymes (*i.e*., GGPPSs from *Taxus canadensis*, *Abies grandis* and *Nicotiana tabacum*) to form an active heterodimeric enzyme catalyzing GPP formation [43,46]; even heteroexpression of *A. majus* GPS.SSU in tobacco resulted in an increase of monoterpenoids and drastic effects due to deficiency of GGPP [46]. It is not surprising that plant GGPPSs can serve as the catalytic subunits of GPPS, because the short-chain prenyltransferases, excepting GPPS.SSUs, show a high level sequence similarity to one another, some of which tend to display dual (or adventitious) activities, producing products with more or fewer C5 (IPP) unit(s) than the main product [47]. For example, the homodimeric PaIDS1 which has strong sequence identity to other conifer GPPSs and GGPPSs is a bifunctional GPP/GGPP synthase, whose catalytic properties and reaction mechanism resemble those of conifer GGPPS, except that significant quantities of the intermediate GPP are released [48]. Since no other form of GPPS had been discovered, it seemed that it is the heterodimeric GPPS that is responsible for the formation of GPP in tuberous of *R. glutinosa*. Only a few GPPS.SSUs have been characterized, and due to the divergence of their sequences [45], we cannot identify a GPPS.SSU homologue from the 454 assembly. As mentioned above, a hetero-GPPS.SSU can interact with endogenous GGPPSs and form functional GPPSs. However, whether *R. glutinosa* has only specific genes for GPPS.LSU, or it can also recruit GGPPS genes for this purpose, has yet to be determined. A comparative analysis of gene expression profiles would be helpful for understanding their roles in this plant. In order to achieve this aim, two GGPPS genes, *GGPPS1* and *GGPPS2*, which belong to different mRNA sequences based on blast analysis, one *FPPS*, together with the only *GPPS* identified in this study were selected for real-time PCR (qRT-PCR) analysis. The result showed that the four genes have very different expression patterns. It is interesting that the expression of *GPPS* displayed a flower specific profile; its expression level is much lower in tuberous roots than in flowers. This result showed that this GPPS.LUS would be responsible for the flower GPP biosynthesis, as the flower is one of the major monoterpenoid producing tissues in plants; the floral scent is dominated by monoterpenoids [49]. In contrast to *GPPS*, FPPS, *GGPPS1* and *GGPPS2* showed higher expression levels in tuberous roots (Figure 4). The relative high expression level of *FPPS* in tuberous roots can be due to the needs of steroid and triterpenoid biosynthesis in tuberous roots as described previously. As for the two putative GGPPS genes, *GGPPS1* was highly expressed both in flowers and tuberous roots, and *GGPPS2* was mainly expressed in tuberous roots. A further analysis showed that the deduced amino acids of *GGPPS1* showed high identities to *Humulus lupulus* GPPS.LSU (67%, 88/132) and *A. majus* GPPS.LSU (67%, 89/132), while *GGPPS2* has low identity with *H. lupulus* GPPS.LSU (32%, 25/78) and *A. majus* GPPS.LSU (34%, 26/77). As both flower and tuberous root are rich in monoterpenoids, which respectively secrete or storage monoterpenoid compounds (previous researches had shown that iridoids are accumulated in tuberous roots), these results showed that the possibility of GGPPS1 coupling with *R. glutinosa* GPPS.SSU does exist to from a functional GPPS, though more experiments are needed to support this speculation. Anyway, the data explored here will provide useful information for understanding the iridoid biosynthesis.

## 3. Experimental Section

### 3.1. Plant Materials

The plants of *R. Glutinosa* cultivar 85-5 were grown in a greenhouse using natural light. The vegetative tissues were harvested two months after sprouting, while flowers were harvested from the second year plants in May of 2010.

### 3.2. cDNA Library Construction and Sequencing

The total RNA was extracted as previously described [50]; then RNA samples were treated with DNase I (TaKaRa, Dalian, China). The mRNA was isolated from total tuberous root RNA using an Oligotex mRNA Mini Kit (Qiagen, Hilden, Germany) and then was converted into first-strand cDNA using SMART cDNA synthesis protocol (Clontech, USA) with minor modification: the 3′ SMART CDS Primer II A was substituted with a BsgI site containing poly(T) primer (5′-AAGCAGTGGTATCAACGCAGAGTACT(20)VN-3′). The first-strand cDNA was synthesized into double-strand cDNA and amplified by LD PCR using KOD-Plus-ver2.0 DNA polymerase (Toyobo, Japan). The double-strand cDNA was purified and treated with BsgI to reduce the poly (A/T) tails. The resulting cDNA was fragmented and subjected to sequencing on a 454 GS FLX Titanium platform.

### 3.3. Sequence Assembly

All 454 reads were filtered to remove poly (A/T), low quality sequences and those shorter than 50bp using the SeqClean program. Resulting sequences and quality files were assembled using CAP3 with default parameters.

### 3.4. Functional Annotation and Metabolic Pathway Analysis

The annotation of unique putative transcripts was based on sequence homology using basic local alignment search tool (BLAST) 2.2.17 software. The unique sequences were searched against the UniProt database, the Nr database, the KEGG database, the COG database and the Nt database (*E*-value < 1 × 10^−5^). Annotations of unique sequences used for GO classification were acquired by BLASTX searching of the UniProt database. GO terms were assigned to all well-annotated sequences by performing Uniprot2GO program. The unique sequences were assigned to special biochemical pathways according to the KEGG standards using BLASTX. To reduce the redundancy, each sequence that had BLAST hit in the Nr database was given a unigene ID according to the best homologue they were aligned to; sequences aligned to the same homologue shared the same ID.

### 3.5. Quantitative Real-Time PCR

A total of 0.5 μg of DNase I-treated total RNA was converted into single-stranded cDNA using a Prime-Script 1st Strand cDNA Synthesis Kit (TaKaRa, Dalian, China). The cDNA templates were then diluted 20-fold before use. The quantitative reaction was performed on a CFX96 Real-Time PCR Detection System (Bio-Rad, Singapore) using SYBR Premix Ex Taq™ (TaKaRa, Dalian, China). PCR amplification was performed under the following conditions: 30 s at 95°C, followed by 40 cycles of 95 °C for 15 s, 60 °C for 30 s and then 72 °C for 20 s. The gene expression of was normalized against an internal reference gene *TIP41*. All primers used in this study were listed in Table 3. Three biological replicates were performed for each gene.

## 4. Conclusions

Although knowledge of the molecular base of the seoiridoid pathway is well established in TIA producing plants, little molecular information on the iridoid pathway is yet known. To explore the transcriptome of *R. glutinosa* and identify genes involved in the iridoid biosynthesis, the 454 pyrosequencing technology was used in this study to generate substantial transcriptome sequence data. Based on *de novo* assembly and bioinformatic analysis, all genes involved in terpenoid backbone biosynthesis were identified in the 454 assembly; more significantly, the presence of secoiridoid pathway gene homologues in *R. glutinosa* implied that the early steps of the iridoid pathway share the same as those of the secoiridoid pathway. This study also gives a first insight into the gene expression patterns of four prenyltransferases. The putative transcriptome information and iridoid biosynthetic gene explored in this study will provide a significant contribution towards understanding the *R. glutinosa* iridoid biosynthesis and may help us to enhance rehmannia quality by genetic engineering.

## Supplementary Materials









## Figures and Tables

**Table 1:** Summary statistics of functional annotation for *Rehmannia glutinosa* sequences in public databases.

Database	No. of matches	Percentage
Nr	25,700	43.7%
KEGG	25,259	42.9%
UniProt	25,468	43.3%
COG	5,701	9.7%
Nt	22,427	38.1%
Total	29,053	49.0%

**Table 2:** The candidate genes involved in ridoid biosynthesis identified in *R. glutinos*.

Enzyme name	Enzyme code	Number of unigenes in 454 library	Number of reads in 454 library
acetoacetyl-coenzyme A thiolase (AACT)	2.3.1.9	5	9
3-hydroxy-3-methylglutaryl-coenzyme A synthase (HMGS)	2.3.3.10	5	32
3-hydroxy-3-methylglutaryl-coenzyme A reductase (HMGR)	1.1.1.34	10	185
mevalonate kinase (MK)	2.7.1.36	3	14
phosphomevelonate kinase (PMK)	2.7.4.2	2	2
diphosphomevalonate decarboxylase (DPMDC)	4.1.1.33	4	19
1-deoxy-d-xylulose-5-phosphate synthase (DXS)	2.2.1.7	6	13
1-deoxy-d-xylulose-5-phosphate reductoisomerase (DXR)	1.1.1.267	4	36
4-diphosphocytidyl-2-*C*-methyl-d-erythritol synthase (CMS)	2.7.7.60	1	1
4-diphosphocytidyl-2*C*-methyl-d-erythritol kinase (CMK)	2.7.1.148	1	2
2-*C*-methyl-d-erythritol 2,4-cyclodiphosphate synthase (MECS)	4.6.1.12	4	4
1-hydroxy-2-methyl-butenyl 4-diphosphate synthase (HDS)	1.17.7.1	4	7
1-hydroxy-2-methyl-butenyl 4-diphosphate reductase (HDR)	1.17.1.2	3	7
isopentenyl diphosphate isomerase (IDI)	5.3.3.2	6	17
farnesyl diphosphate synthase (FPP)	2.2.1.10	4	19
geranyl diphosphate synthase (GPPS)	2.5.1.1	1	3
geranylgeranyl pyrophosphate synthase (GGPPS)	1.3.1.74	9	21
geraniol synthase (GES)	3.1.7.3	1	2
geraniol 10-hydroxylase (G10H)	1.14.14.1	1	2
cytochrome P450 reductase (CPR)	1.6.2.4	5	58
10-hydroxygeraniol oxidoreductase (10HGO)	1.1.1.255	6	97
Total number		85	550

**Table 3:** The parameters used for Real-Time PCR analysis.

Gene	Primer sequences (forward and reverse)
*GPPS*	5′-GGGAAAACTGCTGGGAAGG-3′
	5′-TTAGCCAGAGCAACCAAAGGA-3′
*FPPS*	5′-CAGAGGAAAGCCTTACTATGTGGAT-3′
	5′-TGAACAATGCGGCGGTGA-3′
*GGPPS1*	5′-TTGCCCTGTATGGACAACGA-3′
	5′-CGCCGATAACACGAACGATT-3′
*GGPPS2*	5′-AGGGCACAGGCTAAGAAAGAAT-3′
	5′-CCTCTATCAACAGCATAATCAACAAAGT-3′
*TIP41*	5′-ACGCCTCGGATTTCTCATTC-3′
	5′-TCCAGCCGCATAGAGCATC-3′
