# Supplementary material for: Transcriptome Analysis Reveals Putative Genes Involved in Iridoid Biosynthesis in Rehmannia glutinosa
Source: Int J Mol Sci. 2012 Oct 23;13(10):13748–63. doi: 10.3390/ijms131013748 (PMC3509546; doi:10.3390/ijms131013748)
Supplement: Supplementary file 2 [file ijms-13-13748-s002.pdf]

>Contig8783 acetoacetyl-coenzyme A thiolase[2.3.1.9]
CCCAATACAGTTGTCTGCACTACTGTCAACAAAGTCTGTGCTTCTGGAATGAAAGCAACT
ATGCTGGCGGCACAAAGTATCCAGTTGGGTATCAATGATGTTGTCGTGGCTGGTGGCATG
GAAAACATGTCTAATGTCCCAAAGTACCTGCAGAGGCAAGAAAAGGATCTCGACTAGGA
C
ATGATAC
>Contig16773 acetoacetyl-coenzyme A thiolase[2.3.1.9]
CAGCATCAGCATATCCTGTGANCTTTGTCAATGACTTGCAGTCCAAGCTTCAGAGCCTTT
TCTCTACTTACTAAAACAAGAGCCGCAGCACCATCGCTTATGCTAGAAGCATTCCCAGCT
GTGACAGTACCACCAGTTTCCTTAAAACTTGGTCTTAGCTTCCTCAACTTTGCAGCATCA
AACTTTCCTAGACCTTCATCCTTATCTACTAATGTGGATGGTTTTCCTCTTCCTCCAGAC
ACTTCCACTGGGACAATCTCCCATGCAAAGGCACCACTATCTTGAGCGGCAATTCCACGT
TCAAAGCTTTGAACTGCATAATGTCCTGCTCCTCTCTGGTAAC
>GLRWUMC01DYX3S acetoacetyl-coenzyme A thiolase[2.3.1.9]
AGCGCCAGAATTATTCACAACTGCTCCAGCACTTGCAATCCCCAAAGCAATTTCAAATGC
TGGTTTAGAAGCATCTAAAATAGACTACTATGAAATCAATGAAGCCTTTGCGGTGGTTGC
TCTTGCAAATCAGAAGCTATTGGGTCTTAGACCCGGAAAAAGTGAATGTACATGGTGGAG
CTGTATCACGTGGGGCATCCTC
>GLRWUMC01A5Q0S acetoacetyl-coenzyme A thiolase[2.3.1.9]
TGTTTTCAAATCTAAGTACCACAATTTTGATGGACTAAGGAAGATGAAAAATAAGAGACA
TTTCATCTAAATTAGAAGATGGTACAGACAAATGCACTCCACTCAGGTGTTCATGTCTTC
AATGCTGCTAACAACCCGGCGGGATAATGTGTATCTGTGGCAACGTCCATCAATTGAAAG
AAATCTACGATAGAGACGCCAATCAGTAGAATCGGACCGAAAAGTGAAAAATATTACAG
A
AGCTCTAAGACTAGTGCTGAAGCACCTCCGCCTCCATTGCAAACACCACCAACTCCATAT
TTTGCATTTTTCTGCCTCAATACCCCCAAAAGTGTGACCAAGATACGAGCTCCACTACAA
CCTAAAGGATGTCCCAATGATACAGCTCCACCATGTACATTAACTCTTTACCGGATTAAC
GACCCAATA
>GLRWUMC01DNONL acetoacetyl-coenzyme A thiolase[2.3.1.9]
TTGGCCTAAATTTGCACTGAGAACATTTCCAAAGAAGACTTCTTGGACGAGAGATGGATG
TACACCCGCCCTCTTAAGAGCACATGCAATAGCTATAGAACCAAGCTTTGTAGCAGGCAA
AGATGAAAG
>GLRWUMC01CIAYW HMGS[2.3.3.10]
TGTATTGGGGATACCTGCACCTAACGCTGCCTGTCTGGCTGGAGCCTGTCCCAGGTTTGC
ACTGAGTACATTTCCAAAGAAAACTTCTTCAACAAGTGATGGATCAATATTTGCTCTCTT
TAGAGCACTCTCAATAGCAACAGATCCAAGCTTGGTTGCTGATACTGATGAAAGGGAACC
AAGGAAGCCACCCATAGGCGTTCGGGCAACACCAACGATGCAAACATCTCTGGGCTTAAT
GAGTCCGCTGCTGCTGCTGGAGCCAT
>Contig1861 HMGS[2.3.3.10]
CCATGAAGCTGATGGAGCATAGGTATGGAGGCAAGGACTTCAAAACAAACAAGGACTGT
A
GTCTCTTGCAACAGGCACATACTATCTCACTGAAGTGGATTCCAAGTACCGAAGATTCTA
TTCCAAGAAGGTTATTGAGAATGGCACTCTTGCCAATGGGCACTGAAGATTGTTTCACCA

CAATATGATGCCCATGAAGATGGAACTTATTACTTAGGAAAATTAACTGTGTTTTGTTTG
GCTCAAACTTTTCTAGTTTCTTTGGCAGATAGATTTTATATGCAGTTTCATTTGAATTCA
CATTTAGGTAAAAGAGATCATGGCTGTATAGCCTGTATTTTACATCTAGATGAATTTGGT
TTAGCATCTTGTCCTAGTGGCACCCCACAA
>Contig2867 HMGS[2.3.3.10]
ATAAGTGTAGATGGCTGCACCTTAGCATCGTAAAACGGCTTGGCTACTTGTTGAGTCGCC
TTCTCAAGATCGCGACTTTGGTAGCTTTCCTCATTGGTTAATGACGAGAAAGGTGACAGC
TTCTCTTTAGCAGCCTCATCAATGGAGCTGGCACTGCTCAAAAAATCATTGAATAACAAT
CTGGCGAAGCTCTTCTGTACAAGCTTGTTATATGGAGAATGGAATACAAAGTACTCAGCA
TCATTGACCGAAAACTGCTTGCCCTCCAGCTTCTGATACTTGTCACAGAAGCCTTTAGTA
ACAGGAATCCAGTGCCATTAGATAACAAGTCTGAGAAAGCTTGCCATCGACAACTGGATA
TTCACTGGCAAGGTCAGGCTTGTAAAAATCATAAACATGAGCCATATGACTAGCCCTAAG
CTTGCTTTCGAAATAAATAGGAGCATTTGGTCCTATCAGCATGGCAATAGCCGCAGCTCC
ACCAGTTGGCCTAGCTGGTCCCTCGGCGTAGACCGCACTGTCTGTGCAGACAACAAGCCC
ATA
>Contig8401 HMGS[2.3.3.10]
ATCAAAGTAGATGGCTGCACCTTTGCCTCATAAAATGGTTTTGCAACTTGTTGGGATGCC
TTCTCAAGGTCGCGACTTTGGTAGCTTTCGTCAATGGTTAATGATGAAAATGGAGCCAGC
TTTTCTTTAGCAGCCCCATCAATGGAACTGGCACTCCTCAGAAAATCATTGAATAGAAGT
CTAGAGAAGCTCTTTTGTACAAGCTTGTTATATGGAGAATGAAATACAAAATAGTCAGCA
TCAGCAACTGAAAACTGCTTTCCCTCTAGCTTCTCATACTTATTACAGAAGCTCTTAGTA
ACAAGAATCGAGTGCCATGAGTAACAGTCTGAGACAGC
>Contig12553 HMGS[2.3.3.10]
TATGGGCTTGTCGTCTGCACAGACAGTGCGGTCTATGCCGAGGGACCCGCTAGGCCTACT
GGAGGGGCGGCAGCCATTGCCATGCTAATAGGGCCAAATGCTCCTATTGCCTTCGAAAGC
AAACTCAGGGCGAGCCACATGGCTCATGCTTACGATTTTACAAGCCTGATCTTGCCAGTG
AATATCCCGTTGTTGATGGCAAGCTGTCTCAGTACTTGTTACCTCATGGCACTCGTA
>Contig12993 HMGS[2.3.3.10]
CGAAACAAGTGGGGAACATGTATACTGCATCGCTCTATGCTGCATTTGCATCTCTCCTCC
ACAATAAAAATAGCACTCTGGCCGGGCAAAGGGTGATATTGTTTTCCTACGGCAGTGGTC
TATCAGCCACCATGTTTTCTCTCCATTTTAACGAGGGGAACATCCTTTAGCCTCTCCAAC
>Contig19562 HMGS[2.3.3.10]
ATCCATCCATAAACTACTTTCCACCAATTGACACGGTTAAATAATGCAAGACAAGTACCC
CTCGAAGATTGGCATCAAAAATGTCTTGATCGATTTGCTTGTATCGAGTACAGTCTCACT
TCCAACTTCAAGACGCCCAATCTCCTTCGGATCGACCCCATACTTTTCTAGGAGTGATGT
GACAGCTGTCATACTCATGGAAATGACATCTTCGACCTCGGTACAAAATGCCATGCAATC
TTGGCCAAGCCCAATTGTGTATTTCCCTTTGCTTGCTCCATCATGAGCCTCCAGCACTTC
CTGCTGGATGCAGGTTGGAGGAAAGTAAACTTCCATAGCGAGTATTCCCACATTCTTGGC
CATCTCTTTGTTGTAATCTACTGTTTGAGCAATGAAGGAAAAGGCAGATCAATTAAAGCG
AAAAGTAGTTTTTTCTGAAAAAATCCC
>GLRWUMC01AH3R6 HMGS[2.3.3.10]
AACATGGCAACCGTTATGAATGTTTCGGAGAAAGTTGAAGTCAAGGCATGAGTTCCCGCC

AGAAAATTGTCGAGATCATGAAGCTAATGGAGCACAGATATGGAGGCAAGGACTTTTGT
A
ACGAGCAAAAGACTGTAGTCTTCTTGCACCGGG
>GLRWUMC01AMX1N HMGS[2.3.3.10]
CAAAACAAGTGGGGAACATGTACACCGCCTCACTCTATGCTGCATTTGCATCTCTTCTTC
ACAAACAAACATAGCACTTTGGCTGGGCAGAGGGTGGTACTGTTCTCCTATGGCAGTGGC
CTGTCGGCCACAATGTTTTCTCTTCGTTTAAGCGAGGGTCAGCATCCTTTTAGCCTATCA
AACATTGCAGCTGTAATGAATGTCTCGGAGAAGTTGAAGTCGA
>GLRWUMC01AL96N HMGS[2.3.3.10]
CTACGGCAGTGGTCTATCAGCCACCATGTTTCTCTCCATTTTAACGAGGGGAAACATCC
TTTTAGCCTCTCCAACATTGCAACCGTTATGAATGTTTCGGAGAAGTTGAAGTCAAGGAA
TGAGTTCCCG
>GLRWUMC01CEP2S HMGS[2.3.3.10]
GTCCATCCCACGAACTACTTTCCACCCAATTGACACAGTTAAATAGTGCCGCAGTACCTC
CATAGCACGCATTAAGTTGAGTCAACGCCTTCTANGTCAGTATTTCCGCA
>Contig1093 HMGR[1.1.1.34]
ATGCCAACCGGAATCTGCACGTAACCCACAGGCATTTCGCAACACTGGCCAAGTATAGCA
TCGTAATCGAAGCCATCTAATGGCAACCCCGAGAGGGACTTGCCAGTGGTACGCTGCAAG
GCCTCACGGCGGATGGCGGCAGCACGGCGGCAATCCCCGAGCTTCGACTCGAGGACGTA
T
GAAGGGGTCTTCCCCTCCACCACCGACTTAACAATCTCCTCGTCCTCCTCCGAGGATAGA
GGAAGAACATCACGATCATCAATAATCTTAATCTTATTTGTGGGCCTGGGAGAAGCAATC
TTACAATCATCGGATTGAGGGGCCGCGGCGCAGGGGATGGCACGTGTATCCTCCTTGAGC
ATCAAACCGTCATAATCATTTATTCGTCTTCTTCATCCATAAGGTCTTCGTGGGAAGCGC
GTGGAATAATGAGAGACTGGACAAAACCGATGCCGAAAAAGCCTAGGAGATAGATGAAT
G
AGGCGACGAATGTGACTATGGCGGCGATCTCGGAAAGGGTGACGACGTGGAGAGGGGTG
G
AATTACGGATCTTCTCCCTCCATCTGAGGAGCAGATAATAAACGACGGAGAAGAAGAGA
G
TGAAGAAGACGGCGTTTGTAAGATAGGAGGGGGAGGGGGAGGGCGTCGGAGGCCTTGAG
C
GACTGAGAATCGGAATCATTGACGCGGCGGCGCGAGGGGTTNAATTTCGAGGGTGAACG
A
CTCCTTTNATCGATCGGCGACGG
>Contig1977 HMGR[1.1.1.34]
GGACCTTCACGTATCTGTCACCATGCCTTCTATTGAGGTTGGTACTGTTGGAGGTGGGAC
TCAACTTGCTTCTCAGTCAGCATGCTTGAACCTGCTTGGAGTAAAGGGAGCCAGTAAGGA
GGCTCCAGGATCAAACGCCAGGATGTTAGCCACCATTGTTGCTGGTTCAGTTCTTGCTGC
AGAGCTCTCTCTCATGTCAGCAATTGCTGCTGGGCAGCTTGTCAACAGCCATATGAAGTA
CAATAAGGTACTAAATAAAGATGTACTCCAAAGTTTGATTTTAAAGTGTAGTTATCCCCT
ACTGTAATCTTACTTGT
>Contig3551 HMGR[1.1.1.34]

CTGCTTGGAGTAAAGGGAGCCAGTAAGGAGGCTCCAGGATCAAACGCCAGGATGTTAGC
C
ACCATTGTTGCTGGTTCAGTTCTTGCTGCAGAGCTCTCTCATGTCAGCAATTGCTGCT
GGGCAGCTTGTCAACAGCCATATGAAGTACAATAGGTCTAATAAAGATGTCTCCAAAAGT
TTTGATTTTAAAAGNGTAGTTATCCCCTATGTAATCTTACTTGTCAAAACAATAAACGGT
GCTAACCCCATGCTTTTTGCACTCTCATTACTTAACACCATGTGTGAACTGATGAAAAGT
GTAAACGAAGGGAATACTGGAAAACGTCTTATTTGGTTTTTCATTTCTATTGGTTATATT
CTTTTGTAGTACTTTGTATTTTGCTACTTCCTATGTTATTAAGAAATACAAGCTTGTAAT
CTTCTCTATC
>Contig5874 HMGR[1.1.1.34]
CCACCGGTCAAGATCCTGCACAGAATATTGAGAGTTCTCACTGCATCACCATGATGGAAG
CCGTTAATGATGGAAAGGACCTTCACGTATCTGTCACCATGCCTTCTATTGAGGTTGGTA
CTGTTGGAGGTGGGACTCAACTTGCTTCTCAGTCAGCATGCTTGAACCTGCTTGGAGTAA
AGGGAGCCAGTAAGGAGGCTCCAGGATCAAACGCCAGGATGTTAGCCACCATTGTTGCT
G
GTTCAGTTCTTGCTGCAGAGCTCTCTCTCATGTCAGCAATTGCTGCTGGGCAGCTTGTCA
ACAGCCATATGAAGTACAATAGGTCTAATAAAGATGTCTCCAAAGTTTGATTTTAAAGTG
TAGTTATCCCCTACTGTAATCTTACTTGTCAAAACAATAAACGGTGCTAACCCCATGCTT
TTTGCACTCTCATTACTTAACACCATGTGTGAACTGATGAAAAGTGTAAACGAAGGGAAT
ACTGGAAAACGTCTCATTTGGTTTTTCATTTCTATTGGTTATTCTTTTGTAGACTTTGT
ATTTTGCACTTCCTATGTTATTAAGAAATACAAAAGCTTGTAATCTTCTCTATCTTAAAA
GGCTGAATGTTTCTGCAAACATTTTTGTTTTGCTTGGAATCCTGGTAGAAATTTTGTCTT
GAATATCATTTTATTTTTCC
>Contig7603 HMGR[1.1.1.34]
TTCGTAAAATCGCAGAAAGAAAAATAAAATGATATTCAAGACAAAAATTCTACCAGGAT
T
CCAAGCAAAACAAAAATGTTTGCAGAAACATTCAGCCTTCTAAGATAGAGAAGATTACA
A
GCTTTTGTATTTCTTAATAACATAGGAAGTGCAAAATACAAAGTCTACAAAAGAATATAA
CCAATAGAAATGAAAAAACCAAATAAGACGTTTTCCAGTATTCCCTTCGTTTACACTTTT
CATCAGTTCACACATGGTGTTAAGTAATGAGAGTGCAAAAAGCATGGGGTTAGCACCGTT
TATTGTTTTGACAAGTAAGATTACAGTAAAGGGTAATAAACTAACAACTTTAAAAATACA
AACTTTAGGAGACATCTTTATTAGACCTATTGTACTTCATATGGCTGTTGACAAGCTGCC
CAGCAGCAATTGCTGACATGAGAGAGAGCTCTGCAGACAAGAAC
>Contig8521 HMGR[1.1.1.34]
GGCTCCTCCGCCCCCAAACCCTCTCTTCTTCTTTCTCAGATTATCCCCCATTTCTCTCTG
CGCCACCGCAGCTATGGACGTCCGCCGGAGGCCACCCAAGCCTTCACATACTACTAGACA
ACCTCCTCCGCCTCCTTCCACCTCCGCCGCCGTGGATCACCAATCCTCCTCCCCCAAAGC
CTCCGATGCTCTCCCCCTGCCTCTCTACCTTACCAACGGCATTTTCTCCACCTTTTTTCT
CTGTGGCCTATTTCCTCCTCCACCGGTGGCGTGACAAGATCCGTAACTCTACCCCGCTTC
ACGTCCTCACCCTTTCTGAACTCGCCGAATCCTCTGCCTCATCGCCTCCTTCATCTATCT
CCTCGGTATTTTCGGTATCG
>Contig11827 HMGR[1.1.1.34]

CTTCCCGTCAGCCGCTGCACTGCCTCTCGACGTATTCTCGCCGCCTTAAAGCAGTCCCCG
AGCCTCGATTCCAGAGAATAAGACGGGATTTCACCCGAAACAACACAGTCAACGAGCTTC
TCATCGTCCTGCGGAAGCTCCATGGGTGGCGGCCTGGCCTTGGAAATAACCGGCGACGGT
GGCGGCAACGAGCAATGAATATTCCGATCTTCGTGGAGAATAAACCGCTGATGCTCGTCG
TCGCTCTCGGATTTAGAAATAATGACTGAACGAATCGATACCGAAAATCCGAGAGATAGA
TGAAGGA
>Contig12378 HMGR[1.1.1.34]
CACCGGTCAAGATCCTGCACAGAATATTGAGAGTTCTCACTGCATCACCATGATGGAAGC
CGTTAATGATGGAAAGGACCTTCACGTATCTGTCACCATGCCTTCTATTGAGGTTGGTAC
TGTTGGAGGTGGACTCAACTTGCTTCTCAGTCAGCATGCTTGACCTGCTTGGAGTAAGGN
CCAGTAAGGNGCTCCAGGATCAAACGCCAGGATG
>Contig15936 HMGR[1.1.1.34]
CAGGGCCGCTGTTGTTGGACGGAAGGGAGTACTCTGTTCCCATGGCCACCACGGAGGGTT
GCCTTGTGGCCAGCACCAACAGGGGATGCAAAGCCATCTATGCCTGCGGAGGGGCCACC
A
GCGTTCTCCTAAGGGATGGAATGACGAGGGCTCCCGTGGTCAGGTTCGGAACTGCCAAGA
GGGCTGCAGAATTGAAGTTCTTCCTGGAGGACCCTCTCAATTTCGAAGGGCTTGTCGCTT
GTATTCAACAGTTCAAGCCGATTTGCCAAGCTACAGAGCATCAAGTGTGCCATAGCTGGG
AAAAATCTGTATATGAGGTTCACCTGCAGCACAGGGGATGCCATGGGAATGAACATGGT
G
TCAAAGGGTGTGCAGAATGTCATGGACTTTCTCAACAACGAGTTCCCTGACATGGATGTT
ATTGGCATATCTGGAAACTACTGTTCCGACAAGAAACCTGCCGCTGTGAATTGGATTGAA
GGGCGGGCAAGTCAGTTGTGTGTGAGGCAATAATCAAGGAAGAGGTGGTGAAGAAGTAC
T
CAAGACTGATGTTGC
>GLRWUMC01DXFFE HMGR[1.1.1.34]
GGGCCACAGAGTTGAAATTCTTCTTGGAGGATCCTCTCAATTTCGATACGTTGTGTGTCG
TTTTCAACAAGTCAAGCAGATTTGCAAGACTCCAAGGTATTCAATGTGCTATGCTAGGGA
AAAACCTNTACATTAGA
>GLRWUMC01A8JM3 HMGR[1.1.1.34]
TTAGAGAAGATTACAAGCTTTTGTATTTCTTAATAACATAGGAAGTGCAAAAATAACAAA
AGTCTACAAAAGAATAACCAATAGAAATGAAAAAACCAAATGAGACGTTTTCCAGTATT
C
CCTTCGTTTTACACTTTTCATCAGTTCACACATGGTGTTAAGTAATGAGAGTGCAAAAAG
CATGGGGTTAGCACCGTTTATTGTTTTGACAAGTAAGATTACAGTAGGGGATAACTACAC
TTTAAAATCAAACTTTGGAGACATCTTTATTAGACCTATTGTACTTCATATGG
>GLRWUMC01AYS7Y HMGR[1.1.1.34]
ATTACAAACATTTTAAACTTGTTACTTATGAGGCAAAAAACACTGCCCCCAACAGACAAA
CAATTACAATCACAGCTAATAACAAAAAATTGATCACTAAAAAAATAGACGGGTGGAGG
A
AGAGAAAGGGAAAGAAGCAATACAAACACTTCAAATGCTCGTCAGCTTTCCAGATATCG
A
AACGAGCAGAGCCTCACACGATAATAGTTTGCTCATCTCCTGGCCCTTCATCATAACTCT

ATTTTTTACGCTCATCCCTTTTGCATGCATTTCCACTTCTGTAATCAAGAGGAGCCAATT
TTGGCTATGTCTCTGCTTGATTCGGTTGTATTTCATGTGACTCTTAACGAGCTGGCCAGC
T
>GLRWUMC01B00C6 HMGR[1.1.1.34]
TATGTAAATGGCAGATACAATATTGCTAGCATGAGCATTGAAGCCACCCAGAGCTCCAGC
CATGGCAGAACCAGTGAGGTTCTTGAGCATGTTGAGCTCCACCAAGGAANCAACATCAGT
CTTGAGTACTTTC
>Contig14036 mevalonate kinase[2.7.1.36]
TGTCAATAACTATTAAATAGCCGCAGAAACCAGGTAAACACAATTATATTGAGAACGTAT
GGACGTCACCATGGTTTACAAATCATGGTGAACATAGCTGATTTACTACTCCATACAGTG
CACAAATGTGATAACACCAATGTCTTAAAAGGAATCTTGCTTTTTCATACAACTGAAAAT
AAATAGCATGACAAAATAATCGTACAGACATCAAGAAGTCCGAATCTTACACTTTGCAGA
TGTTACTCCTGAGCTATAATATGTTAGGAAGAACCGCTAAAACTAATTTCCATGCCTCTT
CCACCAATTCCCGCGATCAAACATTGGAACCCACATGATTCTAGCTCCGCAATAAGTTTA
TCCACAACTGTTCCTGATAATAAGGTTGGCAGTAGCGTCAAAACGCAGCCTCCTCCACCA
GCTCCGGTTAGTTTGGTATATAGCTTATATTTTAATGTAGTTCGAATCACAGTTTCGATA
GAAGCGTGGCTGACCCCCATGCATTGGAGTAACCCTTGGTTCATCTCCATGAGTTCTCCT
ATTTTTCCTCTTTCTCAGTGATA
>Contig17421 mevalonate kinase[2.7.1.36]
TTGTAAGGTGAAAAGATAATCCACGGGAAGCCATCTGGGATAGACAATACAGTAAGCAC
A
TATGGAAACATGATAAAATTTAGGTCGGGTGAACTGACACGCATCAAGACAAATATGCCT
CTTAAAATGCTCATAACTAATACAAAAGTTGGAAGAAATACGAAAGCGTTGGTAGCTAGT
GTGTCAGAAAGGACATTGAGACATTCTACTGCTATGGCTTCTGTATTTCATGCTGTCGAT
TCCATCAGCAA
>GLRWUMC01CPPO7 mevalonate kinase[2.7.1.36]
GAAGGTCAGAGCCAGAGCCCCCGGAAAAAATCATACTCGCCGGCGAACATGCAGTGGTT
C
ATGGATCCACCGCCGTCGCAGCCGCTATCGATCTCTATACCTACGTCTCTCTTCGCTTCC
CTACTCCCGCTGATAATGATGATGCGCTGAAACTCCACTCAAGGATATGGCCTTAGTAAT
TTTCTGGCCTAGTT
>GLRWUMC01B13WR 5-phosphomevelonate kinase[2.7.4.2]
GATGCAGTCTTTGCCGTCACCTTGGGGTCTTCAAACAGCAACGTGATCAAAGTTTGGAGT
TCACTCAACGTTCTCGCCTTGCTAGTAACAGAGGATCCTCGTGGGCTTTACTTAGAGAGC
AACGATCCACGAACAAAAGAAATTACGGGTGCTGTTTCTTCAATTCATATTTGAGCGAGC
ATTGATTTTAATACTACTTTTAAACTGGTATTAGCAAATTGTAGTTTTCTGCCATGTCCT
GATATAAGTTGGGAGCAATATTTCTGTTTTGTGCAAGAAAATAAGGAAATAAACAGTTAA
ACACACATGTTGTATTAGATTAAATCTTTTAGCCATTAGTTTGGAGTTAAAATCAAGGGA
TACTGATAACTTTATTCGATGATTCTTGTAAGGCATCTATGTCTATTTACTATGAATT
>GLRWUMC01AYJAU 5-phosphomevelonate kinase[2.7.4.2]
TTTTGCCGTCACCTTGGGGCTTCGAGCAGCAACGTGATCAAAGTTTGGAGTTCACTCAAC
GTTCTCGCCTTGCTAGTAACAGAGGATCCTCGTGGCGTTTACTTAGAGAGCAACGATCCT
CGAGCAACAGAAATTACGGGTGCTGTTTCTTCAATTCATATCTGAGTAGAAATCGATTTT

AATACTACTTTTAAACTGGTATTAGCAAATTGTAGTTTTCTGCCATGTCCTGATATAAGT
TGGGAGCAATATGTTCTGTTTTTGCAAGAAAATAAGGAAATAAACAATTAAACACACCT
GTTGTATTAGATCAAATCTTTTAGCCATTAGTTTGGAGTTCAAACCAAAGGATATTGATA
ACTTTATTCGATGATTCTTGAAGGCATCTATGTCTATTTACTTATG
>Contig701 DPMDC[4.1.1.33]
TTGTGATCTTGATACCCTTCTGCTCATCTTCAACATCACTAGCACGTGACCGAAGTTCCC
TTAAACAATTCTGATATCTACCTCCAGAAAGAGAAACCTCCTTGCCGTTAAGCCACATGC
GGTCGTGGGTAAAAGCGGGGCTGACGGCGACGGAAGTGGTGGTGCAAAGGTGGTCAGGG
T
CCAGAGTAACGCTAATACTATCATTGATGGGAAGAATGAGGTCTTCATCTCTCTTTCCCC
AGTACTTTATCACCGCTATATTCGTCGGCGTCTGTGCCGTCACCGTCAAAACCCATTCGT
CCCGCTTTCCGCTGCCATCCTCCGCCCCAGTCTTCGATTTCTATACCGCAAGAGAATGTA
AGATTGCCTTTATTGCTACTCTGTGAGTGTGTATGAGCTGGATGGGATCCTCCCCCGNGT
ACTCTGCGTTGA
>Contig6218 DPMDC[4.1.1.33]
GACCCCGACCACCTCTGCACCACCACCTCCGTCGCCGTCAGCCCCGCCTTCACTCAGGAT
CGCATGTGGCTCAACGGAAAGGAGGTATCTCTTTCTGGAGGCAGATACCAGAATTGCTTG
AGGGAACTTCGCTCACGTGCTAGTGATGTTGAGGATGAGGAGAAGGGTATTAAGATCGC
A
AAAAAGGACTGGGAGAAGTTGCATGTGCACGTAGTCTCTTATAACAATTTCCCTACTGCT
GCTGGGTTGCGTCATCAGCTGCTGGGTTGGCATGCCTGGTTTTTCCCTGGCGAAGCTAAT
GAATGTAGAAAGAAGATCACAGCAAACTGTCTGCTA
>Contig13845 DPMDC[4.1.1.33]
GACCCCGACCACCTCTGCACCACCACCTCCGTCGCCGTCAGCCCCGCCTTCACTCAGGAT
CGCATGTGGCTCAACGGAAAGGAGGTATCTCTTTCTGGAGGCAGATACCAGAATTGCTTG
AGGGAACTTCGCTCACGTGCTAGTGATGTTGAGGATGAGGAGAAGGGTATTAAGATCAA
C
AAAAAAGGCTGGGAGAAGTTGCATGTGCACGTAGTCTCTTATAACAATTTCCCATGCTGC
TGGGGTTGCGTCATCAGCTGCTGGTTTGGCATGTCCTTGGTTTTTTCCCCTGGCGAAGCT
AATGAATGTAGAAAGAAGATCACAG
>Contig11976 DPMDC[4.1.1.33]
AAACTGATTACTGTCTGCACATGTCAGACAAGCAAAAGCTGGAAAATCACGATTTTTAAT
TGCTTCCTCCATTTGAATTATCCGTTTTGGTACTACTTCCTTTGCTCTATGTTTTATAAG
CATACTGGTTTCAACAGTATCACGCATGCCAGAGGTACTACTCGTTTCCTTCTGTCGTGA
ACTTACCACTGCGATGATAATAACAAGATCATCCCAGTGCTTCTCATCTGCAAGTTGAAC
AGCGATGCTGTCGCTA
>GLRWUMC01APFOT DPMDC[4.1.1.33]
AGAATGGGAGTGACAGCATTGCTGTTCAACTAGCAGACGAGAAGCACTGGGATGACCTT
G
TCATTATCATTGCAGTGGTAAGTTCAAGACAGAAGGAAACAAGTAGTACCTCAGGGATGA
AAGACACAGTTGAAACCAGTATGCTTATAGAACATAGAGCAAAGGAAGTAGTACCTAAA
C
GGATAATACAAATGGAGGAAGACAATAAAAAATCGCGATTTTCCAGCTTTCGCTCGTCTG

ACATGTGCGGACAGTAACCAGTTCATGCAGTCTGCCTGGACTACCTTACCCCCTATTACT
TTTACA
>GLRWUMC01CBEGE DPMDC[4.1.1.33]
TGAAATTAGTACTGCATTTGGCCCAGCATCAAAAGTATAAGCCACCTGAGGTGTTCCTTC
GAAACGATTCCATTTTTCAACGCAGCTGATTATCCTATGGGGATGTGTCATTCATGTAAA
ATATAGGGGGTAAGGTATCCAGGCAGACTGCATGAAACT
>GLRWUMC01ER53F DPMDC[4.1.1.33]
TGCAGTCTGCCTGGATACCTTGCCCCCAATATTCTACATGAATGACACATCTCACAGGAT
AATCAGCTGCGTTGAGAAATGGAATCGTCATGAAGAAACACCTCAGGTGGCTTATACCTT
TGATGCTGGGCCAAATGCAGTACTAATTTCATATAACAGAAAGGCTGCAGCCCTTCTGCT
TCAGAGGCTGCTTTTTTACTCCCCCCTNAGTCAGATACTGATTTGAACAGTTATGTTATT
GGT
>GLRWUMC01DKPYH DPMDC[4.1.1.33]
GTTAGCTACTTTATCTGCACAAGACCTGGGAGAGGTCCTGTGGTACTAACTGATGAAAGT
CGATCTCTTATTAACCCGGAAACTGGGCTGCCCAAGTGAAGAGGACCATGTTTTCAGGTT
TTGAATGTGCTGTTGTTGCTCAGGCAGCCTTTACATTTTTTTGTGTTTTTTAGAGTGTTA
CTGCTATGTCATGGTTCAGTTCCTTTGATGAGTTTTTACTTTCCAGACTGTCGTTTACTT
CTATTTTTACCAAATTAATGCACATTTTGTGTAGACATCTAGTCCAGAATGATAACAAAT
AAATTCGGGTTGTTAGTCTGTTGTTGGACT
>Contig7918 DXS[2.2.1.7]
TGGAGTTCGAAGAGAGAATTTCCATATATGACAGATTGCAGAGTACATTCATCAAACCAC
ATATGCTAATCTTTTATTTGGATTTTCTACTTCAAAGTTAAAACTTTGATTAAAATGACC
ATAATATTGTTTCATTGAAGTTTGAGAGCTTCAATTGGCTTTCCCACGAGCGATAAAACA
GTCCCACAAATATGTCTTGAAGAGAGACCTGCTTCTTCTATCTGATCTTGTGGTNCTCCA
TGGTCTATGTATCTATCTGGAA
>Contig16835 DXS[2.2.1.7]
GACTTGATATAAAACTAGTGAGGATGTTATGCAAGAACCACAAATTTCTGATCACAGTCG
AGGAAGGCTCGATTGGAGGATTTGGAGCACACGTGGCTCAATTCATTGCTCTTGACGGGC
TGCTTGATGCGGGAATTAAGTGGCGGCCCATCACTCTACCCGACAACTACATTGAAGGTG
CATTGCCCCAAGATCAACTTGCTCTTGCTGGCCTAACCGGCAATCACATTGCTGCAACAG
CATTGAATCTGCTTGGTCGAACTCGTGATGCTCTTCTCTTAATGTGCTAGTATCTGATTC
CCGTGTCGATAGTACCATTGAATAGGGTGAGACAAAGGAGAAAGAAATGTGGCGTAATT
A
TTTTGGAAGAGCTAGTTAGAGCAAGGGTAACTTGTCACATATATTGCTTTTGTACAGGTT
AATGTATTATATGTGTGTAAACACATGCCTCTGAGGGCATGCTTTGTGCATGTACATTTG
TTTGTGCTGATGAGTCTCATTATACTGTAAAGTATGTAAACTGATTGTGAAGAGAGGACG
GAGAAAATGTTATT
>GLRWUMC01BX1QW DXS[2.2.1.7]
GCTTGGGAGCTGCAGATATGCTTAAATCATCACACAACATATCAATTACAGTAGCCGATG
CAAGATTCTGCAAACCGTTGGATACTGAACTCATCAGACGGCTGG
>GLRWUMC01DP0O6 DXS[2.2.1.7]
CGGTCATAGATGATAGGCCAAGACTAGCTTCAGATTTCCAAGGGGCAACGGCATAGGAG
C

CGTACTTCCACCTAACAACAAAGGAACACCCCTTGAAATTGGGAAGGGAAGAATACTAA
T
GGAAGGCAGCAAAGTTGCAATACTTGGGTATGGTGCAATAGTCCAACAATG
>GLRWUMC01AKFN0 DXS[2.2.1.7]
AGAACATGAAATAATGATCACTGTGGAGGAAGGTTCAATTGGAGGCTTTGGATCACATGT
TTCTCATTTCTTAAGCCTGAATGGTCTTCTGGATGGACCCCCTCAAGTTGAGATCAATGG
TGCTTCCAGATAGATACATAGACCATGGAGCACCACAAGATCAGATAGAAGAAGCAGGT
C
TCTCTTCAAGCATATTTGNGGGACTGTTTTANCGCTCGTGGAAAGCCAATTGAAGCTCTA
CAAACTTCAATGAAAC
>GLRWUMC01BHEHT DXS[2.2.1.7]
TTCCTTTTAATATCTCGTGCAACTGCCATTCCAAGTCCAGAAGATATGCTGCTGCATCCA
TGCCCCGCTCCGAATGGATCAAATACGCCACGGCATGTATAGCTAGATAAATTGTCTGGT
GTTCTAGCTTTATGCAT
>Contig1462 DXR[1.1.1.267]
TTAGCAATAAAATCATGTGGTTTTAATGACCTATTTAATGGATGGACCATTACAAATGTT
TCACATGTTAAACTTATCTCACTATGAAACAATGACTTTTTGGCCCCCCAATTTTTCTAG
CAAAGATGGCAACTTTAAAACCTATATCTAATGCCAATAAGTCCTTGACTACAATAACAA
AGAAACAAGGGACTTTTAACCAAGCCGACAGCTGCAAATTGGCAGCATAATCGCGTGCC
C
ACAAGTCGTAGTGGACGATTTCCTCAAGCGAAGGCGAGGACACCAATTCTGCTCGGTGCT
TATCGCACGTTAGCTCGACGACTTTGAATATGTCCAGGTAACCAATTTGCTCGTTGATAA
ACATTTCCACAGCTTTCTCGTTGGCTGCACTAAGAACCCCGGTCATGGTCCCACCAGCTC
GTCCAGCAGCATAAGCCAAATCCATTGATGGATATTTCACATTGTCGGGAGCCTTAAATG
TAAGAGAGCCAAGCTTGCAAAGATCAAGGCGAGGCCAAGTAATTTCAGAACAGTAAACT
C
TGTCCGGCCATGATAAGGTGTATAGAATAGGTAAACGCATATCAGGCCACCCCAATTGTG
CTAGTACGGATGAATCCTGTGTCTCAATCATTGAATGTATTATCGATTGGGGATGAATAA
CGATCTCAATATCATCATATTCAGACCCCAAATAGATAGTGAG
>Contig6394 DXR[1.1.1.267]
ATATTTCAGTGCATACAAGGCTTGCCAGAAGGTGCTCTGAGGCGTGTAATTCTGACTGCA
TCAGGAGGTGCTTTCAGGGATTTGCCGGTTAGAAAAATTAAAAGATGTGAAAGTGGCAG
A
TGCTTTAAAGCATCCTAACTGGAATATGGGGAAAAAGATTACAGTCGACTCTGCCACACT
CTTTAACAAGGGTTTAGAAGTTATAGAAGCTCACTATCTATTTGGGGCTGAATATGATGA
TATTGAGATCGTTATTCATCCCCAATCGATCATACATTCAATGATTGAGACACAGGATTC
ATCCGTACTAGCACAAGTTGGGGTGCCTGATATGTCGTTTACCTATTCTATACACCTTAT
CA
>Contig7046 DXR[1.1.1.267]
GGGAAATGTTTATCAACGAGCAAATTGGTTACCTGGACATATTCAAAGTCGTCGAGCTAA
CGTGCGATAAGCACCGAGCAGAATTGGTGTCCTCGCCTTCGCTTGAGGAAATCGTCCACT
ACGACTTGTGGGCACGCGATTATGCCGCCAATTTGCAGCTGTCGGCTGGTAAAAGCCCTG
TTCTTGTATGAGCAAGGACTTATTGGCATTAGAGATAGGTTTTAAAGTTGCCATCTTTGC

TGAAAAAATTGGGGGGCCAAAAAGTCATTGTTTCATAGTGAGATAAGTTTAACATGTGAA
ACATTTGTAATGGTCCATCCATTAAATAGGTCATTAAAACCACATGATTTTTATGTTAAC
CATGTTCTTTGAAGTGTGGATCTAAGCTTACAGAATGGTCAGGTGGGGTTCAAGGGGAAT
ACAGGAAGTGTTAGGAAAATGAATAAATTTTTGTTACAAAGAAAGGTGTACTTATGGTTG
CAAATAATGGAAATGAAATGATGCCAATGTTAACCAA
>Contig7071 DXR[1.1.1.267]
CAATGGACTCTCTTTATAGTCAGTGCATCCATTTTCCTTCCTTTTCTAAAGCAAATCCAC
CTTGGGACTTGAGAAGATTAAGGTTGTAATTCGATTTGGAGGAGTCCAAGAAAGAAAGG
G
TCTTGATTTCAGTTGGAGACAGCATATTCAGAGCCATAGCTTACCACGACACGTAGAAAG
CTGAAGAATCAAAAGCTTGTATATATTTTTCCCTCTCTGTTTTCCCGTTTATTTTCAAGA
ACGAAAGCTAAGTACAACGAACAAGACTGGACTTTTGAGTTATTCTAGGATGGCAAAATA
TAAAGATAGCGATGACCGTCTAGAGAGAGAGAGAGAAAAAGAGAAGAGAGGAGAGAGA
TA
TTGAGTC
>GLRWUMC01B6IXJ DXR[1.1.1.267]
GGAAATGTTTATTAACGAGCAAATTGGTTACCTGGACATATTCAAAGTCGTCGAGCTAAC
GTGCGATAAGCACCGAGCAGAATTGGTGTCCTCGCCTTCGCTTGAGGAAATCGTCCACTA
CGACTTGTGGGCACGCGATTATGCCGCCAATTTGCAGCTGTCGGCTGGTAAAAGCCCAGT
TCTTGTATGAGCAAAGGAACTTAATTAGGACAATTAAGAAGAATAAGGTTTTAAAGTTGC
CATCTTTGCTGAAAAATTGGGGGGCCAAAAGTCATTGTTTCATAGTGAGATAAGTTTAAC
ATGTGAAACATTTGTAATGGTCCATCCATTAAATAGGT
>GLRWUMC01CL6RS DXR[1.1.1.267]
AGCCTATTCCTTCAGCTTGGCCGGGCCGTGCTGTTGTTGAGCCCGGCCGTAAAACTTGGG
AGGGCCCGAAGCCCATTTCAATTGTTGGATCCACTGGCTCCATTGGAACCCAGACGTTGG
ACATAGTTGCTGAAAATCCGGATAAGTTTAGAGTTGTGGCACTTGCAGCTGGTTCAAATG
TGACTCTCCTTGCTGATCAGGTGAGGACATTTAAACCTCAATTAGTTTCTGTTCGAAGTG
AGTCTTTGCTTGAGGAGCTCAAAGAGGCTCTTGCTGATATTGAAGACAAGCCTGAAATCA
TTCCTGGAGAGCAGGGATGATAGAGGTTGCCCGGNATCCAGATGCTGTTACTGTAGT
>GLRWUMC01DDQLM CMS[2.7.7.60]
GCGAATCTTTTGTGGTAAAAACTCTGGACAGAAAAACACTCTGGGAAATGCAAACCCCAC
AGGTTATCAAGCCTGACTTGCTTAAAAAAGGCTTTGAACTTGTTAATAGGGAAGGACTTG
AAGTCACTGATGATGTATCAATTGTGGAGCACCTTAAACATCCGGT
>Contig631 CMK[2.7.1.148]
TTGGTGAGGAACCGTCAATCTACGTAATCATAGAGCATTCGTCGATGAATGATGTGACAA
TAATGTTTTTGGGGTACAAATAAAATTGAGACCATAATGTGGTAGTGCATTTCTTTGAAT
TATAACATCTTCTCAATCCTTATCTTGCAACCAACGATGTGAGACCTGTTTGATCTTACA
ACTTGATACAAAATTCACTCAGTTACTATCGATTCTTCCCTGCTGAACATACTTGCTGCT
GAACTGGGAGCAGTGTACCATTGGTTCTCTTGCCTTGTGAGAAAGGAAGCTTCTGATACA
AATGTATCCTTGTAGGTTTCGTCATCGTATATGAACTGGTGGGGGTCCGGCGAGCCAATT
CCAACTATGGTGCTTCCG
>GLRWUMC01APU1Q MECS[4.6.1.12]
GCTGCAAATACATCTTCAAATTCAGTAATATGATTTCAGTAGCATGTGATGGTAGTACAG

TAATTTTGTAGTTCAGTTGCATCCTACTTGCTCATCAGCTGTAAAAACTAACTGGATCTC
AAAGAGCAGTTTATTATCATAAAATGCTAATGTTGTAAGTACAACAAAAAACNTTTCTCT
ATGGCAAAGTCATGTATTATTATTACTTCCTCACAAGTAGAACAACTGTATGTGCTGCAA
TACTCCTGTTCTCCCCAAGACTGTCGACCTTCTTATGTAGTTTTCGCCTTGAG
>GLRWUMC01B0U4S MECS[4.6.1.12]
GGGAAGCAGAGCCCCAGCCGAAAAGCTGCAACAGCCGCGAAGGCTTTGCCCTTCCGCGT
G
GGCCATGGTTTTGACCTTCACCGGCTCGAGCCGGGATATCCTCTCATCATCGGCGGCATT
AATATTCCTCATGATCGAGGCTGCGAGGCTCACTCCGATGG
>GLRWUMC01DJKT9 MECS[4.6.1.12]
GAAGGAAGCAGTTCGACTAATGCATGAAGCCGGTTATGAACTCGGAAACTTAGATGCTAC
ATTGATTCTCCAAAGACCAAAAGTGAGCCCTCATAAGGAGGCTATCAGGTCCAATTTATG
CAAGCTACTCGGGGCCATCCGTCTGTTGTAAACCTCAAAGCAAAGCTCATGAGAAGGTTG
ACAGTCTCGGGGANAATAGGAGTATAGCTGCGCACAC
>GLRWUMC01AKYW2 MECS[4.6.1.12]
TTCGAATTCAGTAATATAATTTCAGTAGTATGTGATGGTACAGTGATTTTGTAGTTCAGA
TGCATCATCAGCTGTAAAAACTAACTGGATCTCAAACAGCAGTTTATCATCATAAAATGC
TGATGATGTAAGTACAACCAAAACATTTCTGTATGGTAACAAGTCATGTATTATTATTAT
TACTTCCTCACAAGTAGAACAACTGTATGTGCTGCAATACTCCTGTTCCTCCCCAAGACT
GTCAACCTTCTCATGTAGTTTTCGCCTTGAGTATTTAA
>Contig11061 HDS[1.17.7.1]
CTTTTCTCTTATTTCTGCACTAATCTCTTGAAGATCGAATAAAAGTTCTTCCACAGGACG
GGCACGACACATACTCTGTCTTTGTATTTCTCATTCTGCAGCCTTGTAGCAAAATTGAAA
GATGTATTTCTGATGAAATCAAAGTCCTGATCTGGGGCTTCCAATAGGATACCATCTCCA
AGTCCATCTACCAGAAGGGCTCCTGCATTGGTCCCAGCACCAATGACCAAATCGTCCTGT
GAATTTGTTTAGGGAATTGTATATGATG
>Contig15561 HDS[1.17.7.1]
ATTCTCATTTACCTGGTGTTTCGATTGCCATCATGGGTTGCATCGTGAACGGCCCAGGAG
AAATGGCTGATGCAGATTTCGGTTATGTTGGTGGTGCTCCTGGAAAGATCGACCTTTATG
TCGGCAAGACTGTGGTTAAAAGAGCCATTGCGATGGAACATGCAACGGACGCCTTGATCC
AGCTCATCAAAGACCATGGCCGCTGGGTGGACCCACCTGCCGAAGAGTAAGAATAAGTA
C
ATTTCC
>GLRWUMC01BQCCD HDS[1.17.7.1]
TTTTGGCAACTCTGGACTCAATCTTGTTAAGAGAGCTACCAGCAGAAGATGATAAAGATG
TTCGACTAGGTCTCAAACGGTTGATAGACATTAGCATGGGAGTTATAACTCCTCTGTCAG
AGCAGTTGACAAAGCCATTGCCCAATGCCCTCGTTATGGTAACTCTCAATGAATTGTCTA
CTGGTGCTTACAAGCTTCTTCCTGAAGGTACACGTTTGGTAGTCTCTGTGCGTGGCGATG
AATCTTATGAAGAGCTGAAAGTTCTGAAAAGTACCGATGCTACAATGATTCTTCATGACC
TACCATAAACAGAAGAGAAAATTAGCTGAGCTCATGCCGCAAGAAGTTCTCAAAATGTA
G
AGTATTCTCAAGTTTAAATGATGAAGTGCGATCGGAGTGGGACAGAATGCGTACCAAGAC
CAT

>GLRWUMC01C1CU6 HDS[1.17.7.1]
CTTCTTCACCCTCCTTTTGAAGTGGCAATTGACCACTTCTACGCTGGAAGTCAAAATAGC
GTCCATTCTTTTCTTCAAATGGTGCCACTCCTATCTGAAGCTCAGATGCTTTCATACCAA
GGTTAGCCAATCTTCTACAGGGATCTATCTCTTCTTCTGGAGGTTCAGTTAGGGAAACTC
GAATGGTACCACCCAGACCATCCTGCATAATTAACAGAGATCTTGATAGCCACAGTAATA
TGATGAGTAGAAGAAATATAAGAAGAATTCACAATTCCTGATTCCGATGTCTACAAGTAA
TATCCCCAAATGACTCTTCATTAATGGCTCCACAGATACACACATAACAAGGAGAAAAGA
GACAGTGGACAAATATAGGTAAATC
>Contig11608 HDR[1.17.1.12]
GAACGCGGAATCCCATCATATTGGATTGACAGTGAGAAAAGGATAGGTCCTGGAAATAA
A
ATTAGTTACAAGTTAATGCATGGTGAGTTGGTGGAGAAAGAGAACTTTCTACCAGAGGGT
CCTATCACAATTGGCATAACATCTGGTGCATCTACACCTGATAAGGTTGTTGAAGATGTC
CTTAGTAAAAGTTTCGACATAAAGCGTGAGGAAG
>GLRWUMC01EFOTN HDR[1.17.1.12]
GAGAAAAGAATAGGTCCTGGGAACAAAATGTTACAAGTTGTTGCACGGTGAGTTGGTAG
A
AAAAGAGAATTGGCTGCCAGAGGGTCCTATCACAATCGGGATAACATCCGGTGCATCCAC
CCCTGATAAGGTCGTTGAAGATGTTCTTAACAAGGTTTTTGAGATAAAGCGTGAGG
>GLRWUMC01DH9F7 HDR[1.17.1.12]
GGGGGAGAAACAGAGGATATCGGAAAATTAGTTGAGAAAACCCTAGATGCGCAAGTACG
G
GGTTGAAAATATTAACAACCACTTCATAAGTTTCAACACTATTTGCGATGCAACTCAAGA
ACGGCAAGATGCTATGTATAAACTGGTCGAAGAAAAGTTAGATCTCATGCTAGTTGTTGG
CGGTTGGAACTCAAGCAAC
>GLRWUMC01CWTT5 HDR[1.17.1.12]
GGATGGCGATTTCTCTGCACTTTTCTCGTATCTCCACTCCCACCGAGCTCTTCTTGCCGG
AGAAGAGAATAATCCGGCATCGGAAACCGTTCCGCGTTCGATTCTCCTCTGCCGGAGAAG
CTGCCACGTCCTCGTCCGTGGAGTCGTCGGAATTTGACGCCAAGGTGTTCCGCCACAACT
TGACGAGGAGCAAGAATTACAATCGCAAGGGGTTTGGACTTAAAGAGGAAACTCTTGAG
C
AAATGAGCCAAGAATATACAAGTATGGTTCGGTTAAATTGTCGCAATTTTGTATTTTTTC
TTAATTTTTTGTTCTTCGCTTGTGGTTTATGTAAAAGNGAATCAGTTGGAGTTTAATTGT
AGAAAAATTGAAAAGTTGTTTACTTTATTTGCTCGGTGT
>GLRWUMC01DBEDZ HDR[1.17.1.12]
TGGCATAACATCTGGTGCATCTACACCTGATAAGGTTGTTGAAGATGTCCTTGTAAAAGT
TTTCGACATAAAGCGTGAGGAAGCTTTGCAACTGGCATAAAACGTCATCTCAGTTCAGCA
TTGTGCAACTTACTTAAACTATGTCGTAGTTAAGCGTTGCTTTGATTACCTCAAATCTAT
ATGATAATACACTTTAATATTTAGGTCTGGTGG
>Contig674 IDI[5.3.3.2]
GGTTGATGAGAATGACCGTGTCGTTGGCCGATACCAAATACAACTGCCATCTCATGGAAA
AGATTGAATCCGAAAATTTGTTGCATAGAGCTTTCAGTGTCTTCCTGTTTAACTCGAAAT
ATGAGCTATTGCTTCAGCAACGATCCGCAACTAAGGTGACCTTTCCTTTGGTGTGGACAA

ACACCTGCTGCAGTCATCCACTGTACCGAGATTCTGAGCTTATTGAAGAAAATGCTCTTG
GGGTGAGAAATGCTGCTCAGAGGAAGCTGTTGGATGAACTTGGTATCCCTGCCGAAGACG
TCCCAGTTGATCAGTTTACTCCTTTGGGCCGTATGCTGTACAAA
>Contig4861 IDI[5.3.3.2]
GGGCCCTTTTTCACTCTCAATCGCCGCCTCTTCTGCGCTCCCTCAGCCCCCAGATCCGCC
ATGTCAGCCACCATAGGAAAGGCAGAGCCCATCTCCGCCGTCGATGGAGTCGCCGATTCC
GCCATGGACGCCGTTCAAAGGCGCCTCATGTTCGAGGACGAGTGTATTTTGGTGGATGAA
CAAGATCGTGTTGTTGGACATGATTCCAAATACAATTGCCATCTGATAGGAAAAAATGAA
TCAGAGAATCTGCTCCATAGGGCTTTTAGTGTTTTTCTATTTAATTCAAAACATGATTTG
CTCCTTCAGCAACGGTCTGCAACCAAGGTAACATTTCCTTGGTGTGGACAAATACCTGCT
GCAGCCATCCTCTGTATCGTGAATCT
>Contig12429 HDR[5.3.3.2]
CTTTGGGTAGGTATCTGCACCTCTATAGCTTGTGAATAGTTTCCATGTCAGCAGCTTCCT
TTAGAGTCCCATTCTCAACATGATCCCACCACTTGAACAAGAAATTATCAACCACCAATC
TGAACCATGGGGATAGCTTCACACCCTCTTCACCAGCATCTGCTTTTCTTAAAATCTCTT
TCAGATCTTCGCGATTCACATATTTGACATCGGCTACTTCATCTGGATTTGGGTGCACCT
TAACATCGCGGACGATGAATAGAAGATAGTCCAATTCATGCTCTCCCCACTTTCCATCAG
ATGGTGTCTTTGTACAGTACTACCGCCCCAACGTAGTAAACTGGTACAACGTGGGACGTC
GTCAG
>GLRWUMC01EDKUR HDR[5.3.3.2]
GACCGCTGATTCAACCATGGACGCTGTCCAGAGGCGCCTCATGTTTGAAGACGAATGCAT
ATTGGTTGATGAGAATGACCGTGTCGTTGGCCACGATACCAAATACAACTGCCATCTCAT
AGGAAAAGNTTAGAATACCGTAAAATTTG
>GLRWUMC01ARSJV HDR[5.3.3.2]
TGGGGTTTAGAAATGCTGCACAAAGGAAGCTTCTGGACGAACTGGGCATACCCGCCGAA
G
ATGTACCCGTGGATCAGTTTGTTCCTCTGGGCCGGATGTTGTACAAAGCCCCGTCTGATG
GGAAATGGGGGGGANCATGAGCTTGATTATCTGCTCTT
>GLRWUMC01D7L80 HDR[5.3.3.2]
CCATCCGATGGAAAATGGGGAGAGCATGAACTTGATTATCTCCTCTTCATCGTCCGGGAT
GTTAGTGTGCACCCAAATCCAGATGAAGTAGCTGATGTGAAATATGTGAACCAACAACAG
TTGAAAGAACTTTTGAGAAAAGCAGATGCTGGTGAGGAGGGTCTCAAGCTCTCTCCATGG
TTCAGTA
>GLRWUMC01DWZIM HDR[5.3.3.2]
GGGGTGAGAAACGCTGCGCAAAGGAAGCTGTTGGATGAACTGGGCATTTCTGCTGACGA
C
GTCCCAGTTGACCAGTTTACTACGTT
>Contig5255 geraniol synthase [3.1.7.3]
AGACCATTGGAGTGCTTCTTGTGGAGTGTTGGCCTCCTTCCAGAGCCTAAGTATTCTACG
TGCCGAATAGAGCTGGCCAAAACCATAGCCATTCTGTTGGTGATCGACGATATTTTCGAT
ACCTATGGCAAAATGGATGAACTCGTTCTCTTCACTGATGCAATTCGAAGATGGGATCTT
>Contig8108 FPPS[2.5.1.10]
GATTTGAGCACTGCCTGCACGGCTTTGCTAGGATGAGCTTCAATAGAGCTTGTTAGTTTC

TCATAGCTCTTGCTCTCAAACTCCGCAAATGCACCTTGGAGATTAATCTCATTATAGAGG
GCTTTGATTTTTGCAACATCAGCCGGATCTGTCTTTCCATAGTGCTCGAAAAGAATTTTC
TTTTGTTCTTCGCTACAAAGCTCCAGAGCTTTTACGACCAGCCAAGAACACTTGAAATCT
TCAATATCTGTTCCAATCTTCCCGATCTTTTCAGGTTCACCAAAGCAATCTAAGTAGTCA
TCCTGTACTTGAAAGTAGATTCCCATAGCAATAAGCACATCCTTCACGTTCACATGGTTG
TCTAGGTTCTCACCCGCCATGAGCAATGCGCATGCAACTGGAAGGTAAAAGGAGTAGTAG
GCAGTCTTGTACTGAACAATGCGGCGGTGAAGAGGCAATGAATATTTTGACAAATCCTTT
TCTCCTTCAATAGTGGTAATTAAATCTATCATCTGCCCTGATGCCGTTTGAAACTCCACC
TCGTTGAACAAATCGAGCAGATCCACATAGTAAGGCTTTCCTCTGAAGTGATTTTTGAGA
ATTCTAGGGATATGGTTACGAAGCATGATTCCATCATTTACAGCAATCATACCAACCTTA
GGGACTCTAAACCAGCATGGTTGACCACGTCGTGTAGTGAGAATTATCCATAATATCATC
AAGG
>Contig20014 FPPS[2.5.1.10]
GAGAGCTGGCATTTGTTGATTTCTCTTCACTCTCCACTGCGTTCAAATTATTCTACCCTG
TGAACAATATTCCTCAATCCTAAGGTGTGGAGATGGCAAATCTGAACGGAACGACGTCGG
ATCTGAGGGCGACGTTTCNGGGGGTNACTCGGTGCTCAAATCTGAGCTCTTGAATGATC
CTGCTTTCGAAT
>Contig21413 FPPS[2.5.1.10]
AACCGAGGCCTGTCAGTTATTGATAGCTACAAGTTACTTAAAGAAGGAAAAAGACTTAAC
TGAAGATGAAGTGTTTCTAGCTAGCGCTCTAGGCTGGTGCATAGAATGGCTTCCAGGCAT
ATTTTCTCGTACTTCGATAGAT
>GLRWUMC01A2CVF FPPS[2.5.1.10]
CAACCGTGTTGGTTTAGGGTCCCCAAGGTTGGTATGATTGCCGTAAATGATGGAATATTG
CTTCGGAATCACATCCCTAGAATTCTCAAGAAGCACTTCAGAGAAAAGCCTTACTATGTG
GATCTGCTGGATTTATTCAACG
>GLRWUMC01EOJIN FPPS[2.5.1.10]
GATTTCAACACTGCCTGCACAGCTTTGCTGGGATGAGCATCGATGGAGCTTGTTATTCTA
TCGTAGCTCTTGCTTTCATACTCGGC
>Contig17725 geranyl diphosphate synthase large subunit[2.5.1.1]
TTCGAACAAGAAACATAATCCAATTAATTTGACAATCTGAATAGTTTCTTGAAACATGAT
CCAATTGCACAATAAAAACACCAATCAACTATAACAAAATCAATTATCCCTATAAGCAAT
ATAATTAGCCAGAGCAACCAAAGGAGCAGCCTTAACAGAATCAAACCCAAGAAGCTGCT
C
TTGAGCTTCCCTATTCAACTTCTCAGCAAGCTCTCTTGATTTCTCAATTCCAAGAAGCTT
AGGATAAGTTGTCTTATCAGCCACCAAATCCTTCCCAGCAGTTTTCCCCAATTCTTGTGA
ACTCTTAGTAACATCAAGAATAATACA
>GLRWUMC01AL3AR geranyl diphosphate synthase large subunit[2.5.1.1]
GGGATTAGAGCATCTGGAGTTCATCCATGTCCACAAGACTGCAGCTTTATTGGAGGCGTC
TGTGGTTATGGGGGCAATTTTGGGTGGTGCTGATGATGAAGATGTTGAGAAGATG
>Contig1814 GGPPS[1.3.1.74]
TGCTCCAATCCGATATCCAGTCGCCTCCCCTGCCCACCGCATCTCAGATCCGCAACCTGG
CCGGCGACCACCCCCTCCGGCCCAAACAACCGCGCTAATTCGCCGATAACACGAACGATT
TTCTCCGGCGACACTCCTCGTGTCGAATCGGCGATGTGCTCGAACGCGCGGGCGAGAAGC

GCGTACCCAGCCAAAACAGTGACGTTTTCGCCGAAAACCACGTGGTTCGAGGGCCTGCCG
CGGCGGAGATCGTCGTTGTCCATACAGGGCAAATCGTCGTGCATTAGAGACATGGCGTGA
ATCATTTCCAGTGCGCAGGCTGACGGCATCGCCGTGGATTCATCGCCGCCGACGAGCTGA
CACGCCGCCAGGCATACAATAGGGCAGATGCGTTTGCCCCTGATAGCAGAGAGTATCGCA
TTGCTTCGTGG
>Contig6256 GGPPS[1.3.1.74]
TGGAGTACATTCACCTGCACAAGACGGCGGCGTCGGTGGAGGCGTCGGCCGTGGCGGGG
G
CGGTTTTGGGAGGCGCGTGTGAGGAGGAGATCGAGAGGCTGAGAAAATATTCGAGATGT
G
CTGGGCTGATGTTTCAGGTGGTGGATGATATTCTTGATGTCACGAAGAGTTCTGAAGAAT
TGGGGAAAACTAGTGGGAAGGATTTGGTGGCTGATAAAGTTACTTATCCAAAACTGATTG
GGATTGATGAATCCAAAAAGGTATGCTCAGAAGTTGAATAGGGAAGCTCAAGAGCAGCT
T
AATGGGTTTGATCCAGAAAAGGCTGCTCCATTGATTGCTTTGGCGGATTACATTGCTCAT
AGGCAAAAGTGATAATGGGCTTCATCTCCATTAAATTCTCTTTTTCTTTTTTATTATCAA
AATAAAATATTTCCACAAGTTTTTTAATAATAAATAATATGTTATGTTGTCA
>Contig6918 GGPPS[1.3.1.74]
ACGATGTGGCGGAAACCAAGAGGGAACAATGCGTCCCCTGCTAAGATTGCCATGTCCAC
A
CCGAATACGGTGTGGTTGGAAGGTTGGCCTCTACGGGATTGGGAATCATCCATGCATGGT
AAGTCGTCATGAACCAATGAAGCAGCATGAACCATTTCAAGTGCGCAGGCGGTGGGGAA
G
GCGGCGGCGCGGTCGCCACCAAATAGCTCACAGGCGGCAACACACATCACCGGCGGCGC
T
CTCTTTGCGCCTTTAGCCAAAAGCGAATAACGCATGGCTTCGTAGATCCGCTGCGGGTAC
AACACAGGTACTGCATCGTACAAGTTTCTGATCGTA
>Contig11800 GGPPS[1.3.1.74]
GGGGGCAATTTTAGGGGGAGGAACTGATGAACAAGTTGAAAAATTAAGAACATTTGCTA
G
AAAAATTGGGCTGCTTTTTCAAGTGGTGGATGATATATTGGATGTGACGAAATCGTCAGA
GGAATTGGGGAAAACTGCAGGGAAAGATTTGGAGACGGATAAAACAACGTATCCGAAGC
T
GTTGGGACTTGAGAAGGCTAGAGAGTTTGCGGAGAAGTTGAATGAGGAAGCTAAGCTGC
A
GCTGGCGGATTTCGATCGGGACAAGAGGGCGCCATTAGTGGCTTTAGCTGATTATATTGC
TCATAGGCAGAATTAGATTGTT
>Contig13466 GGPPS[1.3.1.74]
ATGAAGCCATGCGCTATTCAGTTCTGGCGAAAGGCGCTAAGAGAGCGCCGCCGGTGATGT
GCGTCGCTGCCGGTGAGCTCTTCGGCGGAAACCGCCTTGCCGCCTTCCCTACAGCCTGCG
CTCTTGAAATGGTTCATGCTGCTTCATTAATTCACGATGACTTGCCATGCATGGA
>GLRWUMC01AYUSU GGPPS[1.3.1.74]

GATTTTGTATCAGATGGTGGATGACATTTTGGAGGCGAAAACGGAGACGGATAAAAAGA
A
GGGTAAGAGCTATGTATCGGTTTATGGAGTCGAAAAGGCCATGGAGGTGGCCGAGGATTT
GAGGGCACAGGCTAAGAAAGAATTGGATGGTTTGGGGAAATATGGTGATAAGGTTCTAC
C
GTTATATCACTTTGTTGATTATGCTGTTGATAGAGGTTTTCATGTTGGGGAGCAATTCAT
TTGATAGCTAAATTGTCTTTGATTTAAATCATGTATTGTATTAGTATTATAAAGACAGTA
GCCAAATGGTTACTGTCAAAAGAGAACATGTTTTATGTAAGATGTATGCATACATATATT
GCTTATGAGTTATGGACATAGGT
>GLRWUMC01B9C9W GGPPS[1.3.1.74]
GTTGCACCGGCAATAATTCAAATGTGGGATTGGATACTCTAGAATTCATACACATACACA
AAACAGCTGCATTACTGGAGGCCTCTGTTGTTTTGGGGGCAATTTTAGGGGGAGGAACTG
ATGAACAAGTTGAAAAATTAAGAACATTTGCTAGAAAAATTGGGCTGTCTTTTCAAGTGT
GGATGATATATGGAT
>GLRWUMC01CO3TB GGPPS[1.3.1.74]
GGACGCATTGTTCCCTCTTGGTTTCCGCCACATCGTAACACAGACCCCGACAAACCTCGT
CCCCGAGTCCCGTCTCCTTCAAGTCATCACGGAGATTGCACGAGCCGTGGGGTCCACCGG
CATGGCCGCCGGTCAGTTTCTAGACCTTGACGACGGTACG
>GLRWUMC01CGPQW GGPPS[1.3.1.74]
GATCCAACGGCTGAGAAATTACGGTCGGGCTGTGGGTGTTTTGTATCAGGTGGTGGATGA
CATTCTTGAAGCGAAAACAGAGACAGAGGAAAACAAAAAGAAGGGTAAAAGTTATGTAT
C
GTTATATGGAGTAGACAAAGCGATGGAGGTGCCGAGGATCTGAGGTCCCAGGCTAAGAA
A
NAGTTGGATGCATTGGAGAAATAT
>Contig14037 G10H[1.14.14.1]
TAGGCGATGAGCTCCTGCACTTTCCTACACCTGAGATGCTGATTCGCGTCGAGGCGGTTG
CCGGAGAACATATTGGAGTTCAGAGCTTTCCGGAGGCTGCGCCACCTGTTGGCCACCGGA
AGCCAGACGACGGAGTATTTGAACTGGTCGTGCGCGTGGATGGCGTCGGGGACAGTCCTG
CTCGAAAACTGGAGGTCCTGTTTTTGCAGAACTTCCTTAGCCATGGCGGCGGAGGAAATC
ACCACCGTGTTTATGAAGCCAAGGCGGAGGCACATTAGGGAGCCATAAGTTTTGGCGAG
A
CGGGCGAGAGATTTGTGGGGTTGATCGCCGAGCAAGTGGAGGCTGCCGATGAGGGGCAG
C
GGCGTTGGGCCGGGTGGGAGCTTTTCCCCCTTGTGGATAGTGTTTGGAGGCCATGGATTA
AGGTCAGAGCAAATAACAGTCCTAGCAGAATTGTGAGGAAATCCATGGCGTGAAAATGG
T
G
>Contig3856 CPR[1.6.2.4]
TGGAAAATAAACGAAAAATAAGACTATATAATTATACATAGGTGCAAAATCAACATAT
G
CGTTTTGTCAGAACCATCTTGCCGGCTCATTAGGAAGATTACAGAGTAACTGTACCTAAC
ATTGTCTCAATAATTATTTGTATATATCATACATATTTTAATGTATAAAGTGCAAGAAC

CAAAAAATCCCTTAATAAACTAGTTTTCGAAAGAATCCCAAAGAACTAACATTACAATAC
TCGACTTCCTTTGCTTGGGGTGGCAACATAAGGCAGCTATTATACTCTGCATATTACCCT
CCTCTGATGATACTTCATAAATTGCATATTATCGATCTCGACAAGTAAAACTTTGGCGAT
TTAAAAACCGCGACCCTTTATTTCTCCGTCAGACGAAATTATCACCACACATCACGCAAG
TACCTTCCATTCATTTGCAGATTCTTCACGAAGCTCTCGGTTTTGGAGTTGTCCAGGGAT
CCCTGTTCTTGCACAATGGTGTGGAGAGTTCGGTGGACGTCACGTGCCATGCCCTTGGCA
TCACCGCATACGTAAACATAGCCTCCTTCGGAGATCATATTCCACACATCTGAAGCCT
>Contig4292 CPR[1.6.2.4]
TATGTGGGGATGCAAAGGGAATGGCCAGAGATGTTCATCGCACCTTGCACACCATTGTCC
AAACGCAGGAAAATGTGGATTCGTCAAAGGCGGAGGCCATTGTGAAGAAACTCCAGATG
G
ATGGTCGGTATTTAAGGGACGTCTGGTGATTGCCTCAGGTCATTGGATGGTTATATTTCC
TTTGCTTTCTAAACCTTGCCTTAAAGAAGTTCTAGCTGGGTTACGAACTAGCC
>Contig16097 CPR[1.6.2.4]
ATTGTCTTTTAGTCGACTTATGCTTCTGACCCAAGTGAAGCTGATCGGCTCAAACATCTT
GCTTCTCCTGCTGGAAAGGAAGAATATGCGCAGTATGTAGTTGCAAGTCAAAGAAGCCTA
CTTGAGGTCATGGCTGAATTCCCATCTGCCAAGCCTCCGCTCGGTGTTTCTTTGCGGCA
GTTGCTCCTCGCCTGCAGCCCAGATTTTATTCTATTTCGTCCTCCCCAAAGATTGCACCA
TCAAGAATCCATGTGACTTGTGCATTAGTATACGAGAAAACACCCACAGGACGAATTCAC
AAAGGTGTTTGCTCGACATGGATGAAGAATGCTGTGCCCGCGGAGGACAGCCTCGATTGC
TCTTCCGCACCAATTTTCGTTAGGACCTCTAATTTCAGACTCCCTGCCGATCCTAAAGTA
CCAATAATAATGATTGGCCCTGGAACCGGTTTGGCTCCGTTTAGGGGGTTTCTTCAGGAA
AGGTTAGCTCTGAAGGAATCTGGAGCCGAGCTTGGTCCTGCCATATTATTTTTCGGATGC
AGGAACAGTAAAATGGACTTCATTTATCAAGATGAGTTGAATAACTTTGTCAAAGCTGGA
GTGATTTCCGAGCTCGTCCTCGCGTTTTCACGCGAGGGACCTACTAAGGAATATGTGCAG
CACAAGATGGCACACTGAGTCGGAGA
>Contig19826 CPR[1.6.2.4]
GATATTTTCCTCTCTCATTCGGTAGAAGTCAGAGTCTCTCCAAACTTACCAACGCTCTCT
CAACAAAGTTCATCAAAACCTTCACGCAAACACCCAAATTCACAAAGAGGGAGAGACTC
T
GATCAACAACACGCTGCGAATTATTATTTGTTTAATTGATTCATTATCCTCTGTCGTTTT
TTTTGGGGGGGAAACAGCTTGGACGCAGTTCAATACTCATGGAGCCCACTTCTGAGAAGC
TTTCGCCGTTCGATTTGATGACTGCGATCTTGAAAGGAGTAAAAATCGACAATTCGAATG
GGTCTGCGGAGGCGGCGCCGCCGGCTGTGGTGGCGTTGCTGATAGAGAACAAGGACCTC
A
TGATGATGATAGCCACCTCTGCCGCGGTGCTCATAGGCTATCTTCTGTTCATGTCATGGC
GGCGAGCAGCCAGATCGGCCGAAAAGAAAGTGGTGGAGCTGCCGAAGCTGGCCGTCCCC
A
AGGCGGCGGCGGAGCCGGAGGAAGTGGATGATGGGAAGAAGAAAGTTACCATATTTTT
G
GGACACAGACTGGCACTGCTGAAGGCTTTGCTAAGGCACTGGCTGAGGAAGCTAAAGCT
A
GATATCAGCAGGCCAAATTCAAAGTGGTTGACTTGGATGATTACGCGGCCGACGATGAAG

AGTATGAGGAGAAGATGAAGAAGGAGACTTTGGCATTCTTCTTCTTGGCCACATATGGAG
ATGGTGAGCCTACCGACAATGCGGCGAGGTTCTACAAATGGTTTACAGAGGGAA
>GLRWUMC01B4IP6 CPR[1.6.2.4]
TGAACCATTTTGTGCTGCACATATTCCTTAGTGGGTCCCTCACGCGAGAATGCGAGGACA
AGCTCAGAAATTACTCCAGCTTCTACAAAGTTGTTCAACTCATCTTCATAAATAAAATCC
ATTTGACGGTTTCTACACCCAAAAATATATGGCAGGACCAAGTTCAGCTCCAGATTCCTT
TAGAGTCTAACCTTTCCTGAAGGAAACCCCTAAAAGGNCCAAGCCAGTACCAGGGCCGAT
CATTATTATTGGTACTTTAGGA
>GLRWUMC01AI9B6 CPR[1.6.2.4]
TGTAGAAATCCTCTGAAAGGAGCCAAACCAGTCCCAGGTCCCACCATGATAATTGGAATA
GAAGGATCAGCTGGCAATTTGAAATTAGATGGCCTAATGAAGATAGGAGCCCAGCTACA
G
GCATTGTGCCTCTCCAAGGGAACTGCATTCTTCATCCAGGTAGAACACACTCTTATGAAT
TCGGCCGGTGGGACTCGCACCATAAACTAGTGCACAGGTCACATGAACACGACTTGGAG
>GLRWUMC01DAMFX CPR[1.6.2.4]
AGGCTTCGGATGTCTGGAATATGATCTCTGATGGAGGGTATGTCTACGTCTGTGGGGATG
CTAAGGGCATGGCACGTGATGTCCACCGAACTCTTCACACCATTGTCCAAGAGCAGGGAT
CTCTGACCAGCTCCCAAGCTGAGGGCATGGTGAAGAATCTACAAACGACTGGAAGGTATT
TGCGCGACGTATGGTGATAATTCCTAAATTTTTGAGAAACATATGGCAATGCTGATAGGA
AAAACATGGGCCGATGACCATTATATCACCAAGAT
>Contig10851 10HG0[1.1.1.255]
CTCGGCGCCGACGCGTTTCTGATTAGCTCCGAGCCGGCGGAGATGCAAGCCGCGGCTGGG
ACCTTGGACGGGATTATCGACACGGTTTCGGCGGCTCACCCGGTTCAACCGTTGTTTAAC
TTGTTGAAGCCGGATGGGAAGCTGATTGTGGTCGGCGCGCCGGAGAAGCCGCTCGAGGT
G
CCGGTGTTCTCGCTGATAATGCAGCGGCGGACGATGGCGGGGAGCATGATCGGCGGGATT
AAGGAGACGCAGGAGATGATTGATTTCGCGGCGAAGCATAATATATTGCCTGATGTGGA
G
ATGATCTCGATGGATTATGTGAATACTGCAATGGAGCGGCTTGCGAAAGCCGATGTGAAA
TATCGGTTTGTGATTGATGTTGCGAATACACTGAAAGCCGAGTGATTGAACTCGGCTTGG
CTTGGCTCGGCGGCATGTACTGAAAGCGTGATTTTTGAATGTGTGGTGTGGAAAATGTGG
AGATTTAGTTGAGAAATATTTACTCTTTTACTTTTTACTGCTTTATATGTTTTATGGATT
GTGTTTTAGTAGTTACTGGAATTGGCTCCTGGATGAATAAAATGTCAGTTTGATAATTAG
TCATTTCAAATAGGCTCCTTTTTGTTGGTGTTGAAATTTTATATTTCAACCATATTTATT
TTTTGCCTTTATGACCATGTTTCCACTTAACAATTTATTAATTCAACTAACCCATATCCC
AA
>Contig12508 10HG0[1.1.1.255]
GGGCTCGACAAGCCCGGGACCCACTTGGGCGTGGTGGGTCTAGGTGGTCTAGGCCATGTG
GCTGTGAAGTTTGCTAAAGCGTTTGGGGCGAAAGTGACGGTGATAAGTACAAACCCTGAT
AAAAAAGAGGAAGCGATCAAGAATCTTGGTGTCGATTCGTTTTTGGTTAGCCGTGATCAA
GATCAGATGATGGCTGCAGCAGGGACCATGGATGGGATATTGGATACAGTTTCTGCTCAT
CATGCTGTGCAACATTACNCAACCTCTT
>Contig10160 10HG0[1.1.1.255]

GTACCATTATTAAGTTTGTTAAAGCCTCATGGGAAGCTTATCTTGGTCGGTGCTCCAGAA
AAGCCCCTCGAGCTTCCCGTTTTCCCCCTGATTATAGGGAGGAAGGGAGTATTTGGAAGT
GCAATTGGAGGAATGAAAGAGACACAAGAGATGATTGATTTTGCAGCCAAGCACAATAT
A
CTACCGGACGTGGAGATGATCCCGATCGATTACGTGAACACCGCGATGGAGCGTCTTTTG
AAATCCGATGTCAAGTACCGTTTCGTGATTGACATTGCGAAATCGTTAAAATCTGAGTAA
GACTGTTAGAATAGTCCTCTCCGAAGTTCGCCTCTTCAAGCGAACACGCCTTTGAATCGG
AATAATTTTTATTTTATTATTGTGTTTATGATACATTTTCATTCATGTTATCGTTCGATC
GATGCTTGAGCATAGTTTCGTTGTCTACTTTAGAATAAGAGAGCTTACATATATGATGGA
TGATCTTGTTGCAAGAATCGATCAACCTATGCGATACTTTGTTCATTGTGTTGTGAAAGT
TAAACATTACTAATGTAAGCCAGTTGTTTGTATTGTTGTATTTGGTGAATTAGTCATGCT
GTGAAATGGGATGATTGCAAAAACTACCCTGGAGGGTTGGTTTGAAA
>Contig11537 10HG0[1.1.1.255]
GTGGTATTTGCCATTCAGATCTTCACATGGTCAAGAATGAGTGGGGTTTCACACAGTATC
CTATTGTGCCTGGGCACGAGATTGTAGGCGAAGTAACTGAGGTGGGTAGCAAGGTCGAA
A
AATTCAAAGTCGGCGACAAAGTAGGCGTTGGATGCATGGTCGGATCGTGCCGCGAATGC
G
ACCAATGCACTAACGATCTCGAGAATTACTGCCCCAAACAAATACTCACGTACAGCGCAA
TGTACACGGATGGGACCATCACGTACGGGGGTTACTCTGATCTCATGGTGGCCGACGAAC
ACTTCGTTGTTCGTTGGCCCAAAAACTTTCCCCTTGATCTCGGCGCTCCTCTCCTTTGTG
CCGGTATTACAACTTATAGTCCGTTGAGGCACTTTGGGCTCGACAAGCCGGGAATGAACG
TTGGCGTTGTTGGGCTTGGGGGGCTCGGTCATGTGGCTGTAAAATTCGCTAAGGCTTTTG
GGACTAAGGTGACGGTCATAAGTACGTCTTTGCGTAAGAAAAATGAAGCGATCGAAAAG
C
TTGGCGCGGACGAATTCTTGGTTAGCGGTGACCAAGAGCAGATGCAGGTAAACTTTTACC
CGAGACATTGTGATTAAGGGATGCAAACACTTAATAAAGT
>Contig10544 10HG0[1.1.1.255]
TCACCAGACGGGCCACCGGCAACCGGGATGTCCAGTTCAAGGTTTTGTACTGTGGTGTCT
GTCATTCGGATATTCACATGATCAAGAACGATTGGGGCTTCGCCCATTACCCGATGATCC
CGGGTCACGAGATCGTCGGCGTCGTCACCGAAGTCGGGTCGAAGGTTCATAAATTCAAGG
TCGGAGACAATGTCGGCGTCGGAGTTATAGTAGACGCGTGCCGGAAATGCGATCAATGTG
TTAACGACCTGGAGAATTACTGTCCCGAACGGATCGACACCTATAACGGCGTTCTACCCG
ACGGGTCCCTCACCTACGGCGGTTACTCTGACATTATGGTGGCTGACGAGGAGTTCGTCA
TCCGCTGGCCGGACAATTTCCCGATGGATAAAGGCGCCCCTTTAGTCTGCGCCGGAATCA
CCACTTACAGTCCGTTGAGATACTACGGGCTCGACAAGCCCGGCCTACACGTCGGGGTCG
CCGGGCTTGGCGGGCTGGGCCACGTGGCGGTCAAATTCCTCAAGGCTTTCGGAACAAAGG
TAACGGTTTTGGACATTTCACCTGATAAGAAAAGTTACGCTATTGATACCCTCGGCGCCG
ACGGCTTTATACTCAGCAATAGGCCGGCTGAAATGGAAGCCGCGGCTGGGACTTTGGATG
GTATTCTGGATTGTGTCTCTGTTGTTCACTCTCTTCAGGGGTTGTTGGATTTGTTGAAGC
CACATGGGAAGCTGGTTGTGGTCGGCTTGCCGGAGAAGCCGCTTGAGCTGCCCTATTTTT
CCGTGACTCTGGGACGGAAAATGATGGGCGGGAGTATGATTGGAGGGCTAAAAGAGACA
C

AGGAGATGATTGATTTTGCTGCGAAACATAATATATTGCCTGAAGTTGAGTTATTCATGG
ATTATATTAATACTG
>Contig15418 10HG0[1.1.1.255]
ATAATCAAAACACATTCACAACTTTACATTAATTGCCCATTTCTTCTGTGTGTTTTTACT
CTGTTTTTTGAGATATTGTAAAGAAAAAAAGTTTGAAAGATTCAATTTTTGATAAATGGC
AAAATCTCCAGTTAAGGCATTTGGATGGGCTGCAAAAGACACATCTGGGGTTCTATCCCC
TTTTAATTTCTCTAGAAGGGCTACTGGTGATGATGATGTACAGATCAAAGTGTTGTATTG
TGGCATCTGTCACTCTGATCTTCACAATATCAAGAATGAATGGGGCTTCTCTCAGTACCC
TATGGTTCCAGGGCATGAGATTGTTGGAGTTGAACAGAGGTAAGGAAAAAAAGTTCACA
A
AATTCAAAGTAGGAGACAAAGTTGGCGTCGGATGCATGGTCGGCTCGTGCCG
>GLRWUMC01CC54O 10HG0[1.1.1.255]
AGAGGTGAAATTCTTGGATTTATGAAAGACGAACAACTGCGAAAGCATTTGCCAAGGAT
G
TTTTCATTAATCAAGAACGAAAGTTGGAGGGCTAAAAGAGACACAGGAGATGATTGATTT
TGCTGCGAAACATAATATATTGCCTGAAGTTGAGGTTATTTCCATGGATTATATTAATAC
TGCCATGAAGAGGCTTGCAAGAGGTGATGTGAAATTTAGGTTTGTTATTGATGTCGGGAA
AACACTTAAAGCCGATTGATTGGACTCGGCGCTGGCTTACGGCGAATCTAGGAAACNGGG
GAGTCTTTTTAGTTAGGGTTATATTCTGTTCTTTCCGTTTTTATTTTCGGTTNTTTTTGT
ATTTTGTAATG
>GLRWUMC01C9OWY 10HG0[1.1.1.255]
TTGAACACAAATTTAAATTTATTCATCGAGGAGCAAATGGATGTAAAAACATAAACCAGC
AAAAAGAAAACAACTCGAAACTTGTTGGCGGCACCAAAAAAAGAGAAAAAAAAAGGAC
TC
CAATCTCTTCAAAGAACCTATTCATCTTTCAATGTCTTTCCCACATCAATTACAAACCTA
AATTTGACATCACCTCTAGCAAGCCTCTCCATAGCAGTATTCACATAGTCCATGGAAATA
ACCTCAACTTCAGGCAATATATTATGTTTAGCCGCAAAATCAATCATCTCCTGCGTCTCT
TTCA
>GLRWUMC01DNEGP 10HG0[1.1.1.255]
CGGCACGATCCGACCATGCATCCAACGCCAACTTTTGTCGCCTACTTTGAATTTTGTGAA
CTTTTTTTTCCTACCTCTGTCACAACTCCAACAATCTCATGCCCTGGAACCATAGGGTAC
TGAGAGAAGCCCCATTCATTCTTGATTATTGGTGAAGATCAGAGTGACAGAT
>GLRWUMC01BDZGO 10HG0[1.1.1.255]
GATACCATCATCACATTGCTTTGATATTTCAGATTCAGATACATTAATTTTTTCAGAACT
AGAGTAAAACAGGTTTCCAGAAATGGACGGATACAGTGAAAGCAGTCGGGTGGGCCGCC
A
GAGACCCGTCCGGATTTCTCTTCCCCTCTCAAGTTCACCAGACGCGCCACCGGCGAGCGT
GACGTGCAATTCAAGGTGTTGTACTGCGGGATCTGCCATTCGGATCTCCACAGCGTAGAA
AACGNGTGGGGATCCGCTCAGTACCCG
>Contig13370 10HG0[1.1.1.255]
GCGGGGAGCCTGATTGGTGGGCTGAAAGAGACGCAGGAGATGATTGATTTCGCGGCGAA
G
CATAATATAGTGGCTGACGTGGAGGTTATTTCGATGGGTTATGTGAATAAGGCGATGGAG

CGGCTTGCTAAGGGAGATGTCAAGTATCGGTTTGTTATTGATGTTGGGAAAACACTCAAA
GTTGAATGATTTAAATCGGGTGGGTTTGGTGAAATTAACGGGATTGAGATTTCCTATTCC
ACTTTTTCTGTTTTACTGTTATTCCATCACATTATATGAATGACAT
>GLRWUMC01CDI8V copalyl diphosphate synthase [5.5.1.13]
TTTCTTACTAAAACTAATCATACTTTGATAATTAATAATTTACACCCACATAACTTACAT
GGTCAGAGTACTTTTTCAAAGAGTACTTTGGCGATGTGGTAGTTTATAGTCCCAGGACTA
AAATACGCCGCATAATAGAAACTCCTAGCAACAGTAAGAAAGTTTTGCTTAGTACTAGAA
TCCAAATCTCCATCAGATTTGGTGATCACTAATTTCACTAGTTCTTGCATCTCTGATTCT
ATTTCAACGGTTGTGATGCATCCCCTATTAGTCATGCACCCATTGCTATCGTGCACCTTA
ANAGTGAGATTCCGATGGTAGCGCCCAAACGCAGCGTTATG
>GLRWUMC01DY909 casbene synthase[4.2.3.8]
TTGGTCAATTAATTGCATATATGTTGGAGAATTAATTATCATGGTTCTAATAAAAATAAT
AAGTATTTCGTCCTATTGTACCAGAAGGTATATGGTGGCCATGTTGCCTGAACACTCGAA
AATGAAGTGCAACAGTGTACAAATCATAGGTTTCATCCTCATAGTTTGTATTAAGATTGA
ATAATTGTTCCAATTTCTTTTCAATTTCATTTTCGTAGTGGTATGATATGCCAAGACGCT
CAAGCGTGTCGATCAGATTGAGAGCATCCACAATGTTAGTAGCAGTAGTTGT
>Contig12137 geranylgeranyl reductase[1.3.1.83]
CATCGCCTTCCAGGAGCGCATCAAAATCCCAGACGAAAAAATGAAGTACTACGAAGACC
T
AGCAGAAATGTACGTCGGAGACGACGTCTCCCCTGATTTCTACGGCTGGGTTTTCCCCAA
ATGCGACCACGTCGCCGTCGGCACCGGCACCGTCACCCACAAGGGCGACATCAAAAAAC
T
CCAGATCGCCACCAGACTCCGCGCCAGCGACAAAATCGAGGGCGGAAAAATCATCCGCG
T
CGAAGCCCATCCCATCCCCGAACACCCCCGGCCGAAACGAGTCCTCGACAGGGTGGCCCT
CGTCGGCGACGCCGCGGGTTACGTCACAAAATGCTCCGGCGAGGGGATATACTTCGCGGC
GAAGAGTGGGCGGATGTGCGCGGAGGCTATGGTGGCTGGAT
>Contig12609 geranylgeranyl reductase[1.3.1.83]
AACTGCCAGAAGACAAAAATGAAATACTACGAAAATCTAGCGGAGATGTACGTGGGAGA
C
GACGTCTCACCTGATTTCTACGCGTGGGTTTTTCCAAAATGCGACCACGTCGCAGTTGGA
ACAGGCACGGTTTGCTCAAAACAAAACATCAAACAGTTTCAAGGCGCAATTAGAAACCG
A
GTCAAACCCAAAATCGAAGGCGGAAAAATCATCAAAGTCGAAGCCCACCCAATACCAGA
G
CACCCCCGACCTACCAGGGTCCGAGGGCGGGTGGCTTTGGTCGGCGACGCCGCCGGCTAC
GTCACGAAATGCTCCGGCGAGGGTATATATTTCGCTGCAAAAAGCGGAAGAATGTGCGG
A
GAAGCAATCGTTCGGGCGTCGGAGAACGGGAAGAAGATGATCGACGAGGCGGATCTGAA
G
AGGGAGTATTTGAGGAAGTGGGACGACGAGTTCTTCTGTACGTTCAAATTCTTGGACGTT
TTGCAGAAGGTTTTCTACGGGAGCAACGCCGGCAGGGAGGCGCTGGTGGAGCTGTGTGGT
AAAGAGTATGTGCAGAGGATGACGTTTTGA

>GLRWUMC01ANYN5 geranylgeranyl reductase[1.3.1.83]
TTCAACTGAAATTATCCAAACTAAATAAATTAAACACTCAAGCATTAATTCTGATAAGGG
GAGTTCTATATTTGTTACGAGGGCTTTGATGAGAGTGGCGCCTTTCTCCTTGGCCCGAAG
CCGGAGGAAATTATCAAGTACTTCACGGCGGAGCATGGAGATGAATTCATGGGGCTTGA
G
TGTTTTCCCAAAATCAACTGTGAGATTTGAGGGTGAGATGA
>GLRWUMC01EL7FQ geranylgeranyl reductase[1.3.1.83]
GACTAACTTCAAGAAAACCTTCGTCGGCCTCCGCCAGTCCTCGCCGGAAAACAATGCCAT
AGTTCTATCCAGACCCACCTCCTCCGCCGTCACCTTACCCCGCCGTAGACTACGTGTAAA
CGCCTCGAAATCTAGCCCGAGAGTCACCGGCCGCAACCTCAGAGTCGCCGTGGTGGGAG
G
CGGACCTGCTGGTGGCTCCGCCGCGGAAACCCTGCCAAGGGCGGCATCGAGACGTTCCTA
ATCGAACGGAAAATGGACAACTGACAAACCCTGCGGCGGCG
>GLRWUMC01EAHG6 phytoene synthase[2.5.1.32]
GTGGGGTCGGCGTTAGTTCTGTATCGACAAATACTGGACGAGATAGAAGCAAACGACTAC
AACAACTTCACAAGGAGGGCGTACGTTAGCAAGCCGAAGAAGTTTATATCTTTGCCAATT
GCCTATGCAAAGGCTCTTGTTCCTCCATCATCC
>GLRWUMC01C38K2 phytoene synthase[2.5.1.32]
TAAGTCTAGTTGGTTATTCTAACGTCTAGAGTACGCGGGCGGCGAGGAAGAGTGTATCTA
CCACAGGATGAATTGGCACAGGCCGGGCTATCAGATAGAAGACATATTTGCTGGGAAAT
A
ACTGATAAATGGAAGAAATTTCATGAAAAAGCAAATCAAGAGGGCAAGGAAGTTCTTTG
A
TGATGCAGAGAGTGGTGTCACAGAGCTCAGCGCAGCTAGTAGAT
>GLRWUMC01C6CFD phytoene synthase[2.5.1.32]
AGGTTTCCTGTCGACATCCAGCCATTCAAGGATATGATTGAGGGAATGAGGATGGACCTC
TGGAAATCGAGATACAAAAACTTTGACGAGTTGTATCTCTACTGTTATTATGTAGCTGGA
ACTGTAGGATTGATGAGTGTGCCGATTATGGGCATAGACCCTGAATCCCAGGCTACAACG
GAGAGTGTCTACAATGCTGCTTTGGCACTGGGGCTCGCAAATCAGTTGACGAACATACTC
AGGGATGTTAGGAGAAGATGCACGTCGAGGAAGAGTGTATCTACCACAGGATGAATTAG
C
GCAGGCAGGGCTATCAGACGAAGACATATTGCTAGGTAAAGTAACCGATAAA
>Contig11662 beta-amyrin synthase[5.4.99.39]
AGAGGCTCGGTTAATACGTAACAACTATCCCAGATCAAGTCTTGTATTAATGGATGAGGA
TAATACATGTCCTCCTTTGCACATAGATGTCGTATGCTCCTCCAATTAATTTCTTCATAA
GGCTGATCATACAATTCTTCTCTTAGTTGTAAAATGAGAGGAGTAATTGGACCTACGAAT
CTCTTGCCGTATAAATACGACATTGGCATGTATACCATTCGACAATAGCACCACTATTTT
TGCTGGATGCAGTGGGGAGAAAAGATGGAAGAAGCCAAAATTCGAGGGGGCAGTTGGGT
T
ACTTCCTGACC
>Contig7597 beta-amyrin synthase[5.4.99.39]
ACTCTAAAGAAAGGGCATGACTTCATAAAGAAATCTCAGGTCAAAGAAGATCCTTCTGGT
GACTTTAGAAGTATGCACCGCCATATTTCTAAAGGATCGTGGACATTTTCAGACCAAGAT

CATGGATGGCAAGTATCCGACTGTACGGCAGAAGGATTGAAGTGTTGCCTTCTATTCTCT
ATGATGCCTCCTGAAGTTGTTGGAAGAAAAAATGGAACCTGAGGGACTCTACAATGCAGT
CAACATATTGCTTTATTTGCAGAGCAAAAATGGTGGTTTAGCTGCATGGGAGCCTGCGGG
ATCCTCGGAGTGGTTAGAGGTAGCTTCTTCTCACAATATTCTTGAACTTTGAAAATATCC
TGGAAATAGGAATATGTGCCGTTGGAAGAAAACCGCTCAAACTTGGTTCATACTGCATGG
GCTCTCCACCGTGCAGCCAAGCTTTTAATC
>GLRWUMC01BMF75 beta-amyrin synthase[5.4.99.39]
CTTCTATGTGAAAACCCCACCCACCGTCTTCATTCTGATGACAATATATGTAACGGAGAA
TTTCACGACGATGTTCTGCTGAAAATACAGTATTGAGATGGCCAGTAATGTACAGAATCA
TGACCAGAGGTGGAAGAAAGAACAATGGACCAGCATTTTCTGCTGGCCAGTGCCCATCAC
TTGCTTGCAAGGCTAGAAAAGAAATGGACAGCTCTCCGCAATGTGGTGGTGGCCGTCTCG
TAGGTTATATCTTCGCCATCGACAACCTTACTTGGGGTATTGTCTGTTTGAATTCTTCTC
ATGCAAGAACTGAAATCGCCAGAGGATATCACTGCTGGG
>GLRWUMC01CUO3B beta-amyrin synthase[5.4.99.39]
CGGGAGAAAGAAATTCAAATCTCCGTGGCAAATGCCTTACGATTCGTCGAACAAAAACA
A
TGGCCTGATGGTTCATGGTATGGTTACTGGGGTAATTTGTTTCCTCTACGGCACATTCTT
CGTGCTAGGAGGATTAGCGTCCGCGAGGGGAAGACGTAACGAAAACAGTGAA
>GLRWUMC01D1O7G beta-amyrin synthase[5.4.99.39]
CTAGAATCCCCGATTATTTATGGGTTGCTGAAGATGGCATGAAAATGCAGAGTTTTGGCA
GTCAACAGTGGGATACAGGTTTTGCTATACAAGCACTGTTGGCAAGTAATATGACTGATG
AAATAGGC
>GLRWUMC01A947I beta-amyrin synthase[5.4.99.39]
GCTACTTATAATTAAATTGCTGAATAATATAAGATTGCAGAAGAGAAATTATTAGGATGT
GGAAGCTCAAGATTGCTGAAGGCCATGGCCCTTATTTGTATAGCACAAATAATTTTGTGG
GCAGACAAATTTGGGAGTATGACCCAAAATGCTGGAACTCATGAAGAACGT
>Contig10148 squalene synthase[2.5.1.21]
GGAACTTGAAGCTCTGTTCGAAATCCTTGAATTTTTTATCGGTGACAACGTTATGAATCA
CAATCCGAACTTTTGATATTGTATGTATATGAATTATTCGTGATTTTGGTGTTGAGCAGG
GTAAAGATGGTGAGTTTGCGTGCGATTTTGAGGCATCCGGATGATTTGTATCCGATGGTG
AAGCTGAAGTTGGCGGCGAGACAGGGCGGAGAAGCAGATCCCGCGGAGC
>Contig16063 squalene synthase[2.5.1.21]
TCTCCGACTCAGTCACAAATGCTTTAGTGCATGTAGAAGACTGCCTAAAGTACATGTCTG
ATTTGCGAGATCCAGCTATCTTTCGCTTTTGTGCAATTCCACAGATAATGGCAATTGGCA
CATTAGCTTTGTGCTACAACAACATTCAAGTCTTCAGAGGTGTTGTGAAAATGAGACGTG
GTCTTACTGCTAAAGTTATTGACAGAACTAAAACTATGTCAGATGTGTATGGAGCTTTCT
ATGACTTCTCTCGTATGCTAAAATCTAAGGTTGACGACAACGATCCTAATGCTAGAAAGA
CCAAGGATAACTTGGAAGCAATCTTGAAAATTTGCAGGGATTCTGGAACCTTAAACCAAA
GGAAATCTTATATTATCCAGAGCAAGCCCCAATATAATGCAACTTTGATTGTTGTCTTCT
TCATCATACTAGCCATTCTTCTAGCATACTTGTCTGCAAGTCGACCATCTAATATGTGAT
TGGTTTTGTGTGGACGACATCATCTATTATCCATTTCTAAGATATAGAGCGAATTCATGT
GTTGGCCATAATCTAGTTAGAGCTGTCAAGTTTTCTGTTGTCCGTATGTAAATTTTACTA
TTTGAGCATTGAAGCCAGTGTACTGTGTACACGTTACCCAGATTACCTAGTTAGAGTTGT

CAAGTTTTCTGTTGTCCGTATGCATCATCATATTGTAAAGCCTACTGCCGAATTACAAGT
TACATGTTGATGCTGTTTGTATCCAATTTTGGCAAGTTTGTTCATTGGCAAAAATGTATG
TTTATGTTCCTGTTTTCTTTAAATAATAATAAAAAAGTAGGTTACTACAA
>Contig8772 squalene synthase[2.5.1.21]
GAAATTTCTCAGGGCCAAACCCAAGCATTTCCAGAGCCAGAAGAAGGTGTAGAATGGGG
A
GTTTGCGTGCGATCTTGAAGCATCCGGATGATTTGTATCCGATGGTGAAGTTGAAATTGG
CTGCCAGAAGTGCGGTGAAGCAGATCCCGGCGGAGCCACACTGGGGATTTTGTTACTCTA
TGCTTCATAAAGTTTCTAGAAGTTTCGGTCTCGTTATCCAGCAGCTCGATACTGATCTTC
GTGATGCTGTATGCATTTTCTATTTAGTTCTTCGAGCACTTGACACTGTTGAGGATGATA
CAAGCATAGCAGCAGAGGTTAAAGTACCTATTCTGATTGCTTTCCATCAGCACATATATG
ACCGTGAATGGCATTTTTCATGTGGTACAAAGGAGTACAAAGTTTTAATGGATGAGTTCC
ATCATGTTTCCACTGCTTTCCTGGAACTTGGACGCGGCTATCAGGAGGCAATTGAGGATA
TTACCATGAGAATGGGAGAAGGAATGGCAAAATTTATTTGCAAGGAGGTAGAAACAGTT
G
ATGATTATGATGAATATTGCCACTATGTGGCTGGACTTGTCGGATTAGGGTTGTCAAAGC
TATTCCATGCTTCTGGAAAGGAAGATCTAGCTTCAGATACTCTCTCCAACTCAATGGGTT
TATTCCTTCAGAAAACAAATATTATTAGAGACTATCTGGAGGATATTAATGAGATACCAA
AGTCACGTATGTTCTGGCCTCGTCAAATTTGGAGTAAATATGTTAACAAACTTGAGGACT
TGAAATATGAGGAAAACTCAGTTAAGGCAGTGCAGTGCCTAAATGACAT
>Contig9786 squalene synthase[2.5.1.21]
CATAACCATACATTTTTGCCAATGAAAAACTTGCCAAATTTGGATACAAACAGCATCAAC
ATGTAACTTGTAGTAATAGGGCAGTAGACTTCACAATATTATGATGCATACGGACAACAG
AAAAGTTGACAACTCTAACTAGGTAATTTGGGTAACGTGTACACTGGCAATGCTCAATAG
TATAATTTACATACGAACAACAGAAAACTTGACAACTCTAACTAGATTATGGGCAAGACA
TGAATTTGCTCTCTAACTTAGAGATGGATAATAGATGATGTCGTCCACACAAAACCAATC
ACATATTAGATGGTCGACTTGCAGACAAGTATGCTAGAAGAATGGCTAGTATGATGAAGA
AGACAACAATCAAAGTTGCATTATATTGGGGCTTGCTCTGGATAATATAAGATTTCCTTT
GGTTTAAGGTTCCAGAATCCCTGCAAATT
>Contig17515 squalene synthase[2.5.1.21]
GAAATTTCTCAAGGCCACACCCAAGCATTTCCGTAGCCAGAAGAAGGTGTAGAATGGGG
A
GTTTGCGTGCGATTTTGAAGCATCCGGATGATTTGTATCCGATGGTGAAGTTGAAATTGG
CTGCCAGAAGTGCGGTGAAGCAGACTCCCGGCGGAGCCACACTGGGTATTTTGTTACTCT
ATGCT
>GLRWUMC01AJPI8 squalene synthase[2.5.1.21]
TGGATAATGTAAGATTTCCTTTGGCTTAAGGTCCCAGAGTCCCTGCAAATTTTCAAGATT
GTTTCTAAGCTGTCCTTTGTCTTTCTAGCATTAGGATCACTTTCATCAACCTTCGATTTT
AGCATAAGAGAAAATCGTAGAAAGCACCAAAGACATCTGACATTGTTTTAGTTCGGTCAA
TAACTTTCGCAGTTAGACCACGTCTCATTTTCACAACACCTCTGAAGACTTCAACGTTGT
TGTAGCACAAAGCTAATGTTCCAATTGCCATGATCTGTGGAATTGCACAAAATCGAAAGT
TGGCGGGATCTCGCAAAGCAGACATGTACTTCAGGCAATCTTCGACATGTGCTAAAGCAT
TTG

>GLRWUMC01ENKR0 squalene synthase[2.5.1.21]
TGACCGTGAATGGCATTTTCTCGTGTGGTACAAAGGAATATAAAGTTCTCATGGATGAGT
TTCATCATGTTTCGACTGCTTTTCTGGAGCTTGGAAGCGGGGTATCGAGGAGGCAATTGA
GGATATTACCATGAG
>GLRWUMC01BN5BQ squalene synthase[2.5.1.21]
ATGTCATTTAGACACTGCACCGCCTTAACTGAGTTTTCCTCGTATTTTAAGTCCTCAAGT
TTGTTAACATATTTACTCCAAATTTGGCGAGGCCAGAACATACGTGACTTTGGTATCTCA
TTAATATCCTCCAGATAATCTCTGATTATATTTGTTTCTGAAGAAATAAACCCATTGAG
TTGGAGAGAGAATCTGAAAGCAGATCTTCTTTTCCAGAAGCATGGAACAGCTTTGACAAC
CCCAGTCCAACAAGTCCGGCCACATAGTGACAATATTCATCGTAATCATCAACTGTTTCT
ACCTCCTTGCATAT
>Contig17464 squalene epoxidase[1.14.13.132]
GTTCCGTTTTAGCTGCCCTTTTCGGGTTTCTCGTTTTATACTATTATGGGTTCTCGAGGA
AAAATGAAAAGAGCTGGTCGACGGAGAATGGGAATAGCACCACCAACACAACCACCATT
G
CTGAAGGAGAATGCGGATCTAGGGGATGGCGGGGATGACGTCATCATAGTCGGTGCTGG
A
GTCGCCGGCGCCGCCCTCGCTCACACTCTTGGAAAGGATGGTCGACGTGTTCGTGTGATT
GAAAGAGATTTGACAGAGCCTGACAGGATTGTTGGAGAACTCTTACAACCAGGTGGTTAC
CTCAAGCTCATTGAGTTAGGATTGGAAGATTGCGTGGAGAAATTGATGCT
>GLRWUMC01EMOZE squalene epoxidase[1.14.13.132]
GCCTATGAGCTTAGCTGCACACTTCTTTGCCGTTGCTGTTTATGGCGTTGGCCGCTTGCT
GCTCCCATTTCCTTCGCCCAAGCGCCTCTTGCTCGGAGCAAAACTCTCTTGAGTGCATAT
GGGATCA
>GLRWUMC01DTXPL squalene epoxidase[1.14.13.132]
GATTTTGTTTTGTACTGCACTCCTCTTATGGTCCCATTTTCTTCAAGAAGAGACGTGACT
GTTCCTTGCTCCAGTTTAACATTTGGAAGAGATGAAGCTTTCTCGCGCATCCTTTGTATG
AAGCGTCCATTATGGAAGCTCCTACCTGATACATCAGAGTGAAATTTTTCCAAAGGATAA
GAAAGACGAGTAGCTTTTCCATCCTTGAATAGAGCATATCCAAACACCCTCTGAGCATCA
ATTTCCTCCACGCAATCTTCCAATCCTAACTCAATGAG
>GLRWUMC01D4VM7 squalene epoxidase[1.14.13.132]
CTTGATCGATAAAAGCATCACGGAGCTCAAATGGAAGCTGAGGAGCCACTTTTGCTATCA
AGTACTTGGCCATGTCTCCATTAGAAATGGAGGGCATTTTGGGGCCAGGAATATCAACCA
AACAGCGGACTTCTGTGCTACTAACAGGATAGAATAAGACGGGCGAAGGATTAGCTAAA
A
TGACATGCCCGTGATTTGGATGTGGAAGCTTGCCGTTCTCTAGGTTGAGGACCATACCAA
CAAAATGACGATACAATTGTTTACC
>Contig4169 obtusifoliol-14-demethylase[1.14.13.70]
GCCGGTCAGCACACGAGCTCGATTACTTCGACTTGGACTGGGGCCTACCTCCTCTGCAAC
CCTAAATATATGTCGGCTGTGTTGGACGAGCAGAAAAAATTGGTGCAAAAGCATGGAAA
T
GAGGTTAACTACGACGTTTTGTCCGAAATGGAAGTTTTATATCGGTCCATAAAAGAAGCT
TTGAGACTCCACCCTCCGTTGATAATGCTTTTGCGAAGCTCGCATAGTGATTTTAGCGTG

ACGACTAAGGAAGGTATTGAGTATGACATTCCGAAGGGACATATTGTTGCCACGTCACCG
GCTTTTGCGAATCGACTCCCGCATATTTAACGAAAATCCGGACGTTTATGACCCTGATTA
GGTTTGCGTCCGG
>GLRWUMC01AO9Z6 obtusifoliol-14-demethylase[1.14.13.70]
TTGTAAAAGCAAATCAATTAAACGAAAAAAAAAAGCAAGAAACAATTTGAATACCTCTG
C
CTCCATAACCATCTGATCAACATAACCTTTGAGCTTATTAACCCTAAGCGCCTCTGTAAA
AAACCTGAACTGCTCCTGTCTAATGGTATAATCCACATCAAACACCACCCCAGGCCCAAA
CGTGGGCACATTGAATTGGTAAACCTCTTGCTGGCTGAGA
>GLRWUMC01BAJ79 obtusifoliol-14-demethylase[1.14.13.70]
GGCTGAGATCGGACTCGGGGGCCTTGAAGAAATGAGCTGAAACTTCTGGACCAATCAAA
A
AGGTTATGTTCTTATTAAGCAATTTTCAGAGTGAACACACTCCCAAGCTTAGGGTACTCC
TCTCTCAGCATCACCACCGGCCCTTTCATGAACCGGATTAAACCGCCGAGAACCGGCCAC
GCGCTCACCGTGGGCGGCAACCGCTTTTTGGATCTGGGCATTATTAAAGCTGATATCAGT
TTTGCAGCCACGTAGAGTGGCTACTAGCAGAA
>GLRWUMC01CETAO obtusifoliol-14-demethylase[1.14.13.70]
AGGGGGACGAGTACGAGATTCCCAAGGGCCATATCGTCGCGACTTCGCCGGCGTTTGCGA
ACCGGCTCCCGCATGTTTTCAAGGACCCGGATTCGTACGACCCGGACCGGTTCGCTGTTG
GGAGGGAAGAGGATAA
>Contig13735 delta14-sterol reductase [1.3.1.70]
GGAGATCGAAGAATCTGCACTTAATAAACATACGGAAGTATCCTCCATGGAACCAGTTTC
GTGTATTCTGCCCATATTTCTTTGTACTTCTGTGCACATCGGGCTTCGTCTCGTCTCTCT
CTCCATATTAGTAGAATGAAAAGGTATATAGGATAAAAATATGGAACTGGGGAACTTGTC
CCACAAGGTAAACTAAAGGATAAAGCTAGCAACAAGTCCCCGAGATAGTTACTATGCCTC
GCAATGCCCCAATAACCAGAAGCAAGCAACTTCCCCCCAACAACCTTAGGAGGTCTACCC
CAGATTAATGCTTTCGGATTCTTTTTGAAATCGTGCTTCTGCTTGTTTGCTCCTCTAAAG
ACCAGATAGCCAATCAGAAAGACAAGACAGTTTGCAACAACAGCAGCAGTCGTCAGCTC
C
ACTTCATTTTTTAGAAGCCACCAACCCTGGATACTGAATGTG
>GLRWUMC01BITNQ delta14-sterol reductase [1.3.1.70]
GGCTCCACTCTCGAGAGCTCGATCGAGAGGAAATCCATGGAGCTGGAGGCCATTCTCTCT
GATCTAACCCCATCTTTGTCATCTGTACTTGGCGATCGCCGGATCTGTTATCCAGGGAAA
GATCGTCCCCGGCGCCGTCCTATCCGACGGCTCTCGTCGCCACTATCGACGCAATGGTGG
TTTGGGGTTCAACTGAATCCTCAATTTTANGGGAGTTGACCTCAAGGTTGGTGGCTTTGG
ACTAACAAAGTGGAATTGACAAGCGCAGTTGCAGTTAACAATATCATT
>Contig13633 C-4 methyl sterol oxidase[1.14.13.72]
CCTTTATATGGGGGTGCTGATTTTCATGACTATCATCACCGACTGCTGTATACAAAGAGT
GGCAACTATTCATCAACTTTTGTGTACATGGACTGGATATTTGGTACTGATAAGGGCTAT
AGAAAACTGAAGGAGCTGAAGAGTGGAGGAGACGAGAAGGCGCCGAAGAAAATGTAAA
AG
TGAACAAGACGTGTTTGGCTGTGGGTTTGTGGTATTGGAGCATCATTTGAACTGGTCTAG
TTTTGGTTCCTCAGCATCTTGTATGCAACTGCATTTTGTGTGCCCAATTGCTGTTACAGC

TAACATTTTGTGTCACGTGGAAAATCAGTTCCTGGCTTGTTATGAATTTTGTGTCCTGTA
ACCACTTCCTATTCATTTTGTTGTGCTCTCTTTGCTAACCAGTCATGGTTTGCATTGAGT
GAAATTCAATATCTGCATGTATGTTGTAAGCTTTCAGAGTCAACTAAATAGATAGTTCCC
TGCTGTTAGTGTATTAGAACTGTAGTCAATGGCAAAAACAATACCTCAATTCACAA
>GLRWUMC01C7059 C-4 methyl sterol oxidase[1.14.13.72]
TTTTACTTGCTCAGCCAACCTGCCCTTTCCAAAAGTATGAATGGAAGTCCCGACAAAAAG
AAGATGCTTTCATGAAGAAAGAAACTTCCAAGACAAGCCAGTTGAAAGTCACTAAAATG
G
GTGATGAGATACATCCAAGCAGACTCGACCATTGAAGCCAGTGGGAAATGACGGACTTG
T
TTTCAGTAGATCTCGGCAGAGAGAGAGTAGAGATACGAGGTGGT
>GLRWUMC01AQ66M cholestenol delta-isomerase[5.3.3.5]
GATGCAGCTGTTGTTACTGTTGAAGCAATTACTGCTGCAATTGAAGGACCAGCTTGCCTT
TTGATTGTGTATGCTATCGGTACAAGGAAGCCATACAGCTACATCCTTCAGCTTGTGGTG
AGCCTCGGCCAGCTCTATGGATGCGCTGTTTACTTGGAACGGCCTACTTGATGGGCATAA
CTTCACTACGAGCCCTTATATTATTGGGCCTATTACATCGGGGCCAACAGCTCGTGGATT
GTCATACCGTCCCTCATCGCAGCCAGGAGCTGGAAGAAGTTAGTAGTGCTTCAGGAAGGG
GAAGGGAAGACTCTGTAGAGTAGTGCTA
>Contig5999 cycloartenol synthase[5.4.99.8]
GAATTTCCCAACATTCTAGTCATTGCTCCTAAAGCTTCATTTTGCAAATGCTTAATTTGG
AATTTCATTTGTCTTTTGCCCCTAAATAGGTTTAAACCAGTTTGAGACGTAGCTTTGCAG
TCCATTGCAGCTTTGGAGAAATCTTCCTGGGCCTGATACACCCGAACCAGATGAAGCTGG
ATCTTCTGTCAGAGAAAGTCAGAGATGAAACCTGAAGTGATAAAATCAAATCGACAGCA
T
CGTAGAGCTTATCAGTCTCTAATGCTTGTCCAGCAACCTCAGATGGCAGCTGTGATAACG
AGGTAAGGGTT
>Contig13862 cycloartenol synthase[5.4.99.8]
TATCCACAACAGGAGATCATGGGGGTCTTCAACAGGAATTGCATGATCACATATGCTGCA
TACAGGAATATATTCCCTATTTGGGCTCTAGGAGAATATCGTACCCGGGGTTCTACAAAC
CCCAATGAATCAGCTGCGGATGAATCTTGAAGTTAGTTTTATGGATTTTGTTACATACCC
TACGGATTTGGATTCGTGTCGTCTTGTAGCTCGTCTCCAATCCGAGTTGGTTTTAGAGTT
CAAAGTCCTAGATATCATCCCAGCTTTGGTTGAAGCAGTTTGGATTTTGTTTCTGCTTTT
ATTATGGTGTTGGTTTTCTCCGCACAATAATTTATTTATAGTTCTATATATTTCTGTACT
TGACTACCTTCCGGCCA
>GLRWUMC01ADBIF cycloartenol synthase[5.4.99.8]
CGTAGCAGCATGTTCTGCACGGCCCTGAATTACGTTAGCTTAAGGCTTCTAGGAGAAGGA
ATGGACGGTGGAGATGGATCCATGCAGAAAGCCAGAACATGGATTCTTGATCATGGTGGT
GCTACCTACATTCCTTCCTGGGGAAAGCTGTGGCTTTCGGTCTTGGCGTGTACG
>GLRWUMC01ALJZF cycloartenol synthase[5.4.99.8]
ACGGTGGATGGGGTCTGCACATTGAGGGCCCAAGCACCATGTTTGGTTCTGTTTTGACCT
ACGTTACTTTAAGATTGCTTGGTGAAGGACCAAATGATGGAGATGGTGCAATGGAGAAA
G
GTCGAGACTGGATTTTGGATCATGGTGGGGCCACTGCAATAACATC

>GLRWUMC01C58ZJ cycloartenol synthase[5.4.99.8]
TATTAGGACTTTTGCTGCACGGTGCAATGGCAAGGGGTCTCTCTCAGCCTGCCCAGCATC
AATGAGAGTCAGCAAAGCCCATCCCGTATTACCACATGAGACCTGTTGCCTTCTAGATTA
GTATACACCTTG
>GLRWUMC01DSG9X cycloartenol synthase[5.4.99.8]
ATATACATTATGAAGATGAAAACACTCGCTACATATGCATAGGCCCTGTAAAACAAGGTG
CTAAATATGCTTTGCTGTT
