# Supplementary figures and images for: Transcriptome Analysis Reveals Putative Genes Involved in Iridoid Biosynthesis in Rehmannia glutinosa
Source: Int J Mol Sci. 2012 Oct 23;13(10):13748–63. doi: 10.3390/ijms131013748 (PMC3509546; doi:10.3390/ijms131013748)

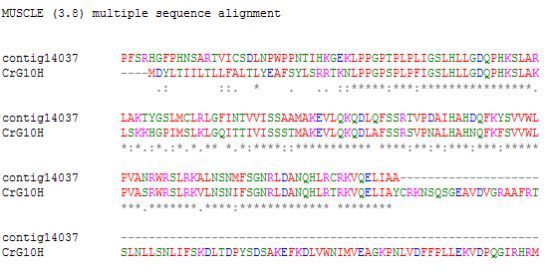

Supplement: Supplementary file 4 [file ijms-13-13748-s004.jpg]
